# Hypoxia induced VEGF secretion promotes resistance to bispecific T-cell engagers

**DOI:** 10.1038/s41392-025-02505-3

**Published:** 2025-12-19

**Authors:** Mengyao Xu, Syem K. Barakzai, Raj Kumar, Irva Veillard, Eugene Kim, Amy Bregar, Eric Eisenhauer, Richard Penson, Sara Bouberhan, Jennifer Filipi, Tina Colella, Tim Bond, Caroline Clark, Lawrence H. Lin, Jinpeng Ruan, Cheng Wang, Xingping Qin, Kristopher Sarosiek, Bo Rueda, Cesar Castro, David R. Spriggs, Oladapo O. Yeku

**Affiliations:** 1 Massachusetts General Hospital Cancer Center, Boston, MA USA; 2 Meigs Division of Gynecologic Oncology, Vincent Department of Obstetrics and Gynecology, Massachusetts General Hospital, Boston, MA USA; 3 Massachusetts General Hospital Cancer Center, Harvard Medical School, Boston, MA USA; 4 Department of Pathology, Massachusetts General Hospital, Harvard Medical School, Boston, MA USA; 5 Department of Obstetrics and Gynecology, Vincent Center for Reproductive Biology, Massachusetts General Hospital, Boston, MA USA; 6 John B. Little Center for Radiation Sciences, Harvard T.H. Chan School of Public Health, Boston, MA USA

**Keywords:** Gynaecological cancer, Tumour immunology, Cancer microenvironment

## Abstract

Bispecific T-cell Engagers (BITEs) are a novel form of immunotherapy that overcome a deficiency of immune checkpoint inhibitors (ICI) by targeting a preidentified tumor associated antigen and redirecting a polyclonal population of effector T-cells against the tumor. High grade serous ovarian cancer is a lethal disease in the recurrent setting and has not been amenable to ICI therapy. MUC16/CA125 is overexpressed in high grade serous ovarian cancer. BITEs targeting the tumor-retained portion of MUC16/CA125 have recently been described and are in early-phase clinical trials. To identify mechanisms of resistance to BITEs, we collected serum, peripheral blood mononuclear cells, and ascites samples from patients with disease progression on MUC16-directed bispecific antibodies. Analysis of these samples showed downregulation of MUC16/CA125, elevated secretion of VEGF, and epithelial-to-mesenchymal transition in tumor cells. Interestingly, hypoxia was determined to be a driver of these changes. These findings were prospectively validated in ovarian cancer cell lines with CRISPR/Cas9 knockout of MUC16/CA125 and VEGF. Peripheral blood mononuclear cells from patients with disease progression were capable of effective cytolysis ex vivo, suggesting that resistance to therapy was primarily tumor driven. Restoration of MUC16/CA125 expression did not restore cytotoxicity in the presence of increased VEGF secretion. Combination treatment with a VEGF inhibitor rescued cytotoxicity in hypoxia-conditioned ovarian cancer cell lines with preserved target antigen expression. Collectively, these data outline a link between hypoxia and the development of resistance to BITEs and posits inhibition of VEGF inhibition as a potentially important therapeutic intervention.

## Introduction

Recurrent ovarian cancer remains an incurable disease. The vast majority of women with recurrent disease develop resistance to conventional cytotoxic therapy and eventually progress and die.^1^ Given this dismal prognosis, immunotherapy is an intense area of investigation for ovarian cancer.^2–5^ Efforts evaluating immune checkpoint inhibitors in patients with recurrent ovarian cancer have been disappointing, both as monotherapy^6–8^ and in combination with other agents.^9–11^ A potential reason for this is the lack of novel tumor associated antigens and immune effector cell engagement in ovarian cancer. Bispecific T-cell engagers (BITEs) are antibodies that consist of two tandemly linked variable heavy and light chains. They have a domain with specificity for a tumor-associated antigen and another domain with specificity for a T-cell antigen (usually the cluster of differentiation 3 (CD3) complex).^12^ Upon administration, BITEs redirect polyclonal T-cells to the tumor. Bound T-cells then differentiate and activate, leading to T-cell mediated tumor-specific cytolysis.^13^ BITEs such as blinatumomab (anti-CD19) and teclistamab (anti-BCMA) have been approved for the treatment of hematologic malignancies; however, developing BITEs for solid tumors has been a more complex endeavor. Hurdles such as target antigen selection, a narrow therapeutic index due to expression of the target antigen in healthy tissue,^14^ and the immunosuppressive tumor microenvironment have limited the rapid development of this treatment modality for solid tumors. Despite these challenges, agents such as tebentafusp (CD3 x gp100) and tarlatamab (CD3 x DLL3) have been approved for uveal melanoma^15^ and small cell lung cancer,^16^ respectively. In uveal melanoma, tebentafusp led to a 49% reduction in the risk of death compared to investigator’s choice therapy. This corresponded to a median overall survival of 21.6 months in patients treated with tebentafusp compared to 16.9 months in the control arm.^15^ Similarly, the phase 3 DeLLphi-304 trial demonstrated tarlatamab’s superiority over standard chemotherapy in the second-line setting. In this randomized clinical trial, patients treated with tarlatamab achieved a median overall survival of 13.6 months, compared to 8.3 months for patients receiving chemotherapy; a 40% reduction in the risk of death. BITEs as an immunotherapeutic modality have the potential to circumvent the limitations of immune checkpoint inhibitors in difficult-to-treat or immunologically cold tumors.

The epitope CA125 or MUC16 was originally described in 1983, is present in 95% of high grade serous ovarian cancer and is highly related to worse clinical outcomes.^17,18^ MUC16/CA125 is a heavily glycosylated member of the mucin protein family. MUC16/CA125 expression is almost universal in high grade serous ovarian cancers making it an attractive potential target.^19^ MUC16/CA125 is composed of 3 major domains and subdomains; a heavily glycosylated large extracellular region including an N-terminal portion, a tandem-repeated domain, and a carboxyl-terminal domain that can be divided into an additional three subdomains composed of an ectodomain, the transmembrane, and a cytoplasmic-tail. A cleavage event in the ectodomain of MUC16 produces a shed portion, known as CA125, that can be measured in the blood for diagnostic and monitoring purposes and a membrane-tethered portion that remains amenable to targeting. While the shed tandem repeat circulates as CA125, the most proximal sequences of the ectodomain, outside the tandem repeat region, are potentially superior targets for therapeutics. Expression of the MUC16 ectodomain is very limited on normal tissues outside the Mullerian tract. Immuno-histochemistry testing of panels of human adult and fetal human tissue demonstrate MUC16 ectodomain presence in Mullerian structures, ovarian malignancies and lobular (but not ductal) carcinoma of the breast.^20^ It is also present on the corneal surface epithelium.^21^

Generation and validation of a bispecific tandem-linked single-chain variable fragment directed to the retained portion of MUC16/CA125 (ectodomain) has been previously described by our group^22^ and others.^23^ In the report by Yeku et al.,^22^ MUC16/CA125 bispecific engagers were more effective in combination with either anti-PD-1 immune checkpoint inhibitors or anti-VEGF antibodies in tumor-bearing mice. In particular, the combination with anti-VEGF antibodies led to significant reductions in ascites and peritoneal disease burden, leading to prolonged survival in tumor-bearing mice compared to mice treated with the bispecific monotherapy or the anti-PD-1 combination. MUC16/CA125 BITEs are currently in phase 1/2 multicenter clinical trials (NCT03564340, NCT04590326) and preliminary results have been reported,^24,25^ including overall response rates of 14.3–18.2%.^26^ What is not known is which mechanisms of resistance may be contributing to disease progression. In addition, the roles of the patient’s effector T cells, antigen expression, and the tumor microenvironment in the development of resistance to BITEs have not been fully elucidated.

In this report, we evaluate mechanisms of resistance to MUC16-directed BITEs using samples from patients whose tumors progressed on MUC16/CA125 bispecific antibody clinical trials. We validate these mechanisms in multiple preclinical ovarian cancer cell line models and critically evaluate therapeutic approaches to overcome resistance.

## Results

### Characterization of tumor samples from patients with progressive disease

**Fig. 1:**
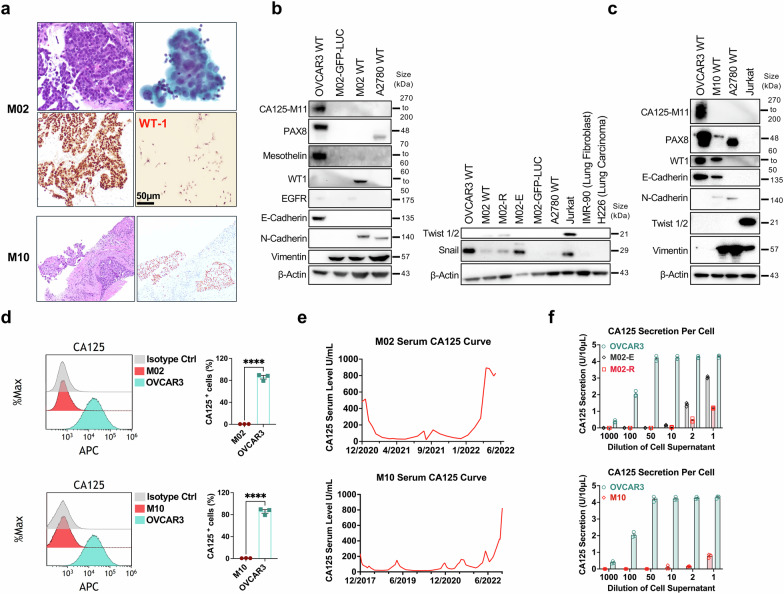
Immunophenotyping of patient samples. **a** M02 top row: Recurrent high grade serous carcinoma (hematoxylin and eosin stain, 400x), and peritoneal cytologic specimen. M02 bottom row: nuclear immunohistochemical expression for PAX-8, and peritoneal tumor cells stained for WT1. M10 top row: Liver core biopsy of recurrent high grade ovarian adenocarcinoma (hematoxylin and eosin stain, 100x), and nuclear immunohistochemical expression for PAX-8. **b** Western blot analysis performed on lysates derived from OC cell lines and cells from OC patient ascites for M02 (M02-E represents M02 cells passed through a fibroblast exclusion column and M02-R represents the cells retained in the column). OVCAR3 cells included as positive control for CA125, PAX8, Mesothelin and E-Cadherin. A2780 cell line included as positive control for N-Cadherin and Vimentin expression. **c** Western blot analysis performed on lysates derived from for M10. Jurkat cells included as positive controls for Twist 1/2 and Vimentin. IMR-90 and H226 cells were used as negative controls for Twist 1/2 and snail. Equal amounts of proteins were separated on 4–12% SDS-PAGE gels and probed for Tumor-associated and EMT-related protein expression. **d** CA125 expression on the M02, M10, and control OVCAR3 cell lines determined by flow cytometry. ***p < 0.001. **e** Serum CA125 trend over time for patient M02 (top), patient M10 (bottom). **f** In vitro CA125 secretion of cultured OVCAR3, M02-E cells, M02-R cells and M10 cells as measured by ELISA. Equal numbers of cells were plated, and the supernatant was collected after 72 h in culture

To evaluate the mechanisms of resistance to MUC16-directed bispecific T-cell engagers (BITEs), we consented patients with platinum resistant ovarian cancer who had progressed on a MUC16- BITEs clinical trial to our tissue collection protocol. Ten patients provided blood samples for analysis, but only two patients (M02, M10) had accessible tumor samples via ascites. Narrative treatment history for M02 and M10 are provided in the supplementary materials. Both patients had high grade serous ovarian cancer. We initially focused on tumor analysis. M02 and M10 samples were morphologically consistent with tumor cells (Fig. 1a). We did not detect CA125 expression on M02 and M10 cells by western blotting (Fig. 1b, c). Western blot analysis of tumor lysates from M02 was positive for WT1 expression (Fig. 1b), while M10 showed Pax8 and WT1 positivity (Fig. 1c), consistent with immunohistochemistry analysis from both patients’ original specimen. Transduction of M02 primary tumor cells with a GFP-luc vector led to selection of cells that no longer expressed WT1 (Fig. 1b). These modified cells were discarded since they no longer represented the parental ascitic tumor cells. Because of the role of epithelial-to-mesenchymal (EMT) transition as a mechanism of immune escape,^27^ we evaluated EMT markers in M02 and M10. Both M10 and M02 tumor cells expressed markers consistent EMT transition such as N-cadherin and vimentin for M10, and vimentin and snail for M02 (Fig. 1b). Full western blot images are shown in Supplementary Fig. 9. The cell lines OVCAR3 (ovarian), A2780 (ovarian), Jurkat (T-cells), IMR-90 (lung) and H226 (lung) were used as controls for our EMT markers. We hypothesized that EMT transition and downregulation of the MUC16/CA125 target could be a potential mechanism of resistance in these cells; indeed, we found decreased CA125 expression in both M02 and M10 tumor cells (Fig. 1d) despite clinically increased CA125 in both patients (Fig. 1e). We verified very minimal CA125 shedding from M02 and M10 tumor cells by ELISA (Fig. 1f). Taken together, ovarian cancer cells derived from patients with progression of disease on MUC16/CA125-directed BITEs showed decreased levels of CA125 expression and expression of EMT markers.

### Characterization of peripheral blood mononuclear cells from patients with progressive disease

**Fig. 2:**
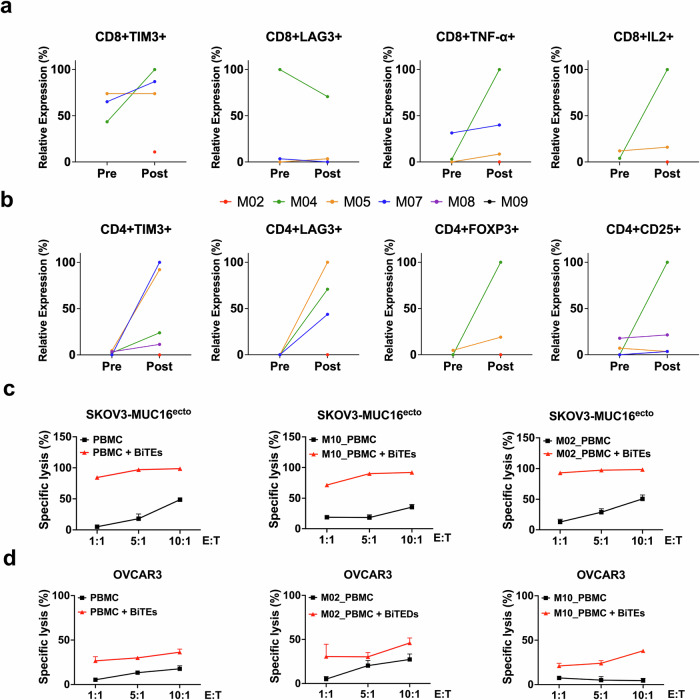
Patient CD8 and CD4 immunophenotyping and functional analysis. Flow cytometry analysis was performed on paired peripheral blood mononuclear (PMBC) cells from pre-treatment and post-progression samples from patients M04, M05, M07, M08 and M09. **a** CD8 and **b** CD4 plots. Relative expression displayed as net MFI divided by the greatest value per cohort. Patient values that were zero before and after were not included; M02 had only post-treatment PBMCs available. **c** Co-culture of MUC16^ecto^-BITEs and healthy donor PBMC, M02 or M10 PBMC with SKOV3-MUC16^ecto^ at the indicated E:T ratios. **d** Co-culture of MUC16^ecto^-BITEs and healthy donor PBMC, M02 or M10 PBMC with OVCAR3 at the indicated E:T ratios

Since BITE therapy relies on cytotoxic effector cells, we immunophenotyped paired PBMCs from patients at baseline (immediately prior to cycle 1 of BITE therapy) and upon progression. Paired PBMC samples were only available for patients M02, M04, M05, M07, M08, and M09. The gating strategy for immunophenotyping is shown in Supplementary Fig 1. We found both activated (CD8^+^ TNFα^+^, CD8^+^ IL2^+^) and dysfunctional (CD8^+^ TIM3^+^) cytotoxic T-cells in patients who progressed on therapy (Fig. 2a). Most of the samples analyzed showed an increase in immunosuppressive regulatory T-cells (CD4^+^ TIM3^+^, CD4^+^ LAG3^+^, CD4^+^ FOXP3^+^) (Fig. 2b). Because the immunophenotyping may not necessarily reflect the functional status of these T-cells, we directly tested if the PBMCs collected from patients who had progressed on BITE therapy for tumoricidal effect in vitro. For these experiments, we cocultured PMBCs isolated from healthy donors or post-progression PBMCs from M02 and M10 with MUC16-directed bispecific engagers^22^ and two different ovarian cancer cell lines. At increasing effector-to-target ratios, PBMCs from both M02 and M10 (Fig. 2c), and M04, M05, M07, and M08 (Supplementary Fig. 2) lysed SKOV3 tumor cells engineered to express MUC16 (SKOV3-Muc16^ecto^) and OVCAR3 cells that endogenously express MUC16/CA125 (Fig. 2d). These results indicate that despite the immunophenotyping results and clinical progression of disease in these patients, their PBMCs were still capable of cytotoxic activity against ovarian cancer cells in vitro.

### Patient serum cytokine profiling and effect on ex vivo cytotoxicity

**Fig. 3:**
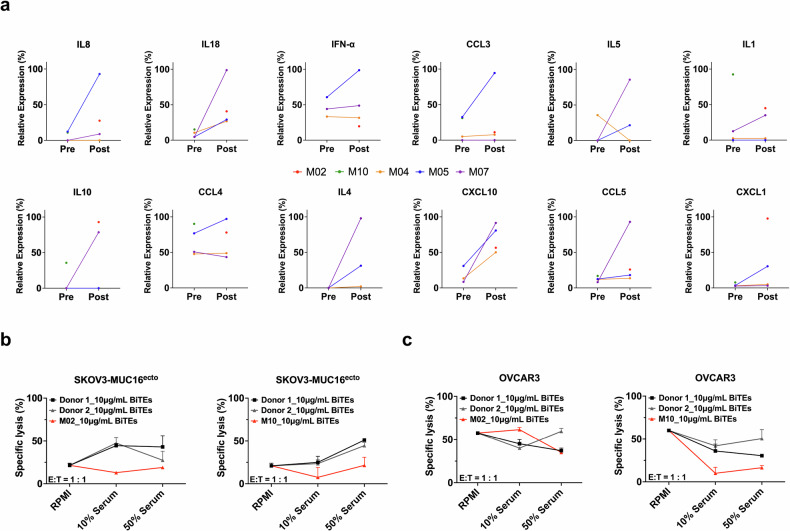
Patient Cytokine Profiling and effect on cytotoxicity. **a** Cytokine and Chemokine secretion in patient serum, pre- and post-treatment. Relative expression plotted as net MFI divided by greatest value per cohort. Patient MFI values that were 0 before and after were not included; M02 had only post-treatment serum available. **b** Cytotoxicity assay involving SKOV3-MUC16^ecto^ cocultured with MUC16-BITEs and Jurkat T- cells in the presence of healthy donor serum, M02 serum, or M10 serum at various concentrations (0%, 10%, 50%) for 48 h. **c** OVCAR3 cells cocultured with MUC16-BITEs and Jurkat T- cells in the presence of healthy donor serum, M02 serum, or M10 serum at various concentrations (0%, 10%, 50%) for 48 h. Specific lysis was determined by luciferase assay

We hypothesized that if PBMCs from patients were still capable of ex vivo cytolysis, the context or humoral microenvironment could contribute to disease progression. We performed multiplex cytokine analysis on paired serum samples from patients M04, M05, and M07 and on single timepoint samples from M02 and M10. Levels of proinflammatory cytokines such as IL18 and IFN-α and chemokines such as IL8, CCL5, CXCL10 and immunosuppressive cytokines like IL4, IL5, IL10 were elevated on disease progression (Fig. 3a). A complete list of profiled cytokines is shown in Supplementary Table 1. Similar to the results with CD8 and CD4 immune profiling results (Fig. 2a, b), this profile seemed to be more in line with generalized inflammation. To evaluate if this cytokine milieu or some other unmeasured component of the patient’s serum was suppressive, we performed ex vivo cytotoxicity assays using MUC16 BITEs cocultured with matched serum and PBMC from 2 healthy donors and matched serum and PMBC from M02 and M10 with both SKOV3-Muc16^ecto^ (Fig. 3b) and OVCAR3 (Fig. 3c) cells. In both ovarian cancer cell lines, serum from patients who had progressed on MUC16 BITEs suppressed cytotoxicity.

### Effect of hypoxia on CA125 expression and EMT phenotype

**Fig. 4:**
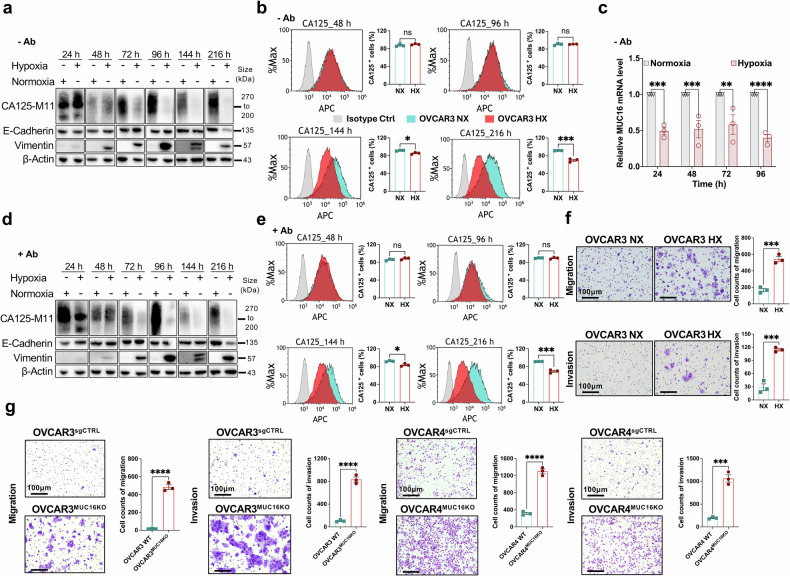
Effect of Hypoxia on CA125. **a** OVCAR3 cells cultured in normoxic (NX) and hypoxic (HX) conditions for 24 h, 48 h, 72 h, 96 h, 144 h and 216 h, and subjected to Western blot analysis for the indicated proteins. **b** Flow cytometry for surface CA125 expression on OVCAR3 cells cultured under NX or HX for the indicated timepoints. Data are expressed as the means ± SEM from three independent measurements, and the differences between the groups were analyzed using the student’s t-test. ns, not significant; *p < 0.05, ***p < 0.001. **c** CA125 mRNA expression in OVCAR3 NX and HX cultured for the indicated timepoints. Data are expressed as the means ± SEM from three independent measurements, and the differences between the groups were analyzed using the Two-way ANOVA. ns, not significant; *p < 0.05; **p < 0.01; ***p < 0.001; ****p < 0.0001 was considered to indicate a statistically significant difference. **d** OVCAR3 cells cultured in NX or HX for the indicated timepoints in the presence of anti-MUC16^ecto^ antibodies and subjected to western blot analysis. **e** Flow cytometry for surface CA125 expression on OVCAR3 cells cultured in the presence of anti-MUC16^ecto^ antibodies under NX or HX for the indicated timepoints. **f** OVCAR3 NX and HX cells evaluated for migration (top row) and invasion (bottom row). **g** OVCAR3 and OVCAR4 cells transduced with CRISPR and Cas9 with MUC16 guide to knockout MUC16 expression (OVCAR3^MUC16KO^, OVCAR4^MUC16KO^) and control OVCAR3/OVCAR4 cells transfected with non-specific guide sequence (OVCAR3^sgCTRL^, OVCAR4^sgCTRL^) were evaluated for migration and invasion. For (**e**–**g**), all data are expressed as the means ± SEM from three independent measurements, and the differences between the groups were analyzed using the student’s t-test. ns, not significant; **p < 0.01; ***p < 0.001; ****p < 0.0001

Both M02 and M10 patient tumor samples were derived from ascites. This led us to investigate if there was something about the peritoneal microenvironment that could downregulate CA125 and suppress cytotoxicity. An important and recognized component of the peritoneal ascitic tumor microenvironment is hypoxia.^28^ We modeled this by exposing OVCAR3 cells to hypoxia and noted downregulation of CA125 over time by Western blot analysis (Fig. 4a). This corresponded to decreased surface expression of CA125 after 144 h and 216 h of hypoxia exposure (Fig. 4b). Full western blot images are shown in Supplementary Fig 10. We verified upregulation of HIF-1α under the hypoxic (HX) conditions used (Supplementary Fig. 3a), without significant increase in apoptosis between normoxic (NX) and hypoxic (HX) conditions (Supplementary Fig. 3b, c). Decreases in CA125 mRNA expression were noted as early as 24 h after exposure to HX conditions (Fig. 4c). HX conditions did not universally lead to mRNA downregulation of other mucins such as MUC1 and MUC3 (Supplementary Fig. 4a). Exposure to HX conditions also led to downregulation of E-cadherin and upregulation of vimentin over time (Fig. 4a), consistent with an EMT phenotype. Exposure to HX conditions in the presence of anti-MUC16^ecto^ antibodies similar to the BITE antibody did not accelerate or attenuate CA125 downregulation or EMT markers (Fig. 4d, e). OVCAR3 and OVCAR4 cells exposed to HX conditions also showed increased migration and invasion compared to NX cells (Fig. 4f, Supplementary Fig. 4b, c), consistent with an EMT phenotype. CRISPR-mediated deletion of MUC16/CA125 led to increased migration in OVCAR3 and OVCAR4 (Fig. 4g) cultured under NX conditions.

### Stabilization of MUC16/CA125 expression in hypoxic-conditioned cells

**Fig. 5:**
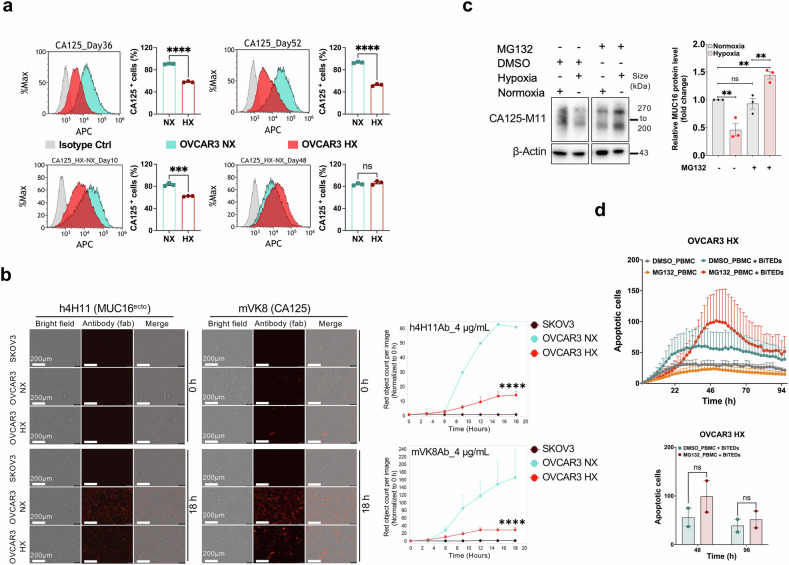
Effects of hypoxia on CA125 expression. **a** Cell surface CA125 expression on OVCAR3 cells cultured under NX and HX conditions for the indicated timepoints. **b** Internalization of CA125 using antibodies against the retained ectodomain (h4H11) or the tandem repeat portion (mVK8) in OVCAR3 cells and SKOV3 (MUC16 negative control) cells under NX and HX conditions. The antibodies only fluoresce upon internalization. **c** OVCAR3 cells treated with MG132 and DMSO diluent under NX and HX conditions and subjected to western blotting for CA125 expression. **d** OVCAR3 HX cells treated with Muc16-BITEDs with or without MG132 and evaluated for cytotoxicity. Data are expressed as the means ± SEM from two independent measurements for (**d**) and three independent measurements for (**a**), (**b**), and (**c**), and the differences between the groups were analyzed using the student’s t-test. ns, not significant; **p < 0.01; ***p < 0.001; ****p < 0.0001

To evaluate dynamic changes in CA125 under HX conditions, we evaluated cell surface CA125 expression on OVCAR3 cells cultured in HX conditions and then transferred back to NX conditions. As shown in Fig. 5a, at day 36 under HX conditions, there is a significant reduction in CA125 expression that persists into day 52 in HX conditions. After 52 days in HX conditions, OVCAR3 cells transferred back to NX conditions for 10 days still had decreased CA125, but by 48 days in NX conditions, CA125 expression was restored. This finding was unchanged in the presence of MUC16/CA125 antibodies (Supplementary Fig. 5a), suggesting that this effect is independent of external anti-CA125 selection pressure. Recovery of CA125 expression in cells exposed to HX conditions after return to NX conditions was also reproducible in OVCAR4 cells (Supplementary Fig. 5b, c). This prompted us to look at CA125 recycling in OVCAR3 cells that had been cultured under NX conditions compared to OVCAR3 cells that had been cultured in HX conditions. We used two different antibodies that recognize different portions of MUC16/CA125. The h4H11 antibody recognizes the proximal retained ectodomain of MUC16 (MUC16^ecto^) and is the similar region recognized by the MUC16 BITE. The VK8 antibody recognizes the tandem repeat domain of CA125.^29^ OVCAR3 HX cells showed significantly decreased intracellular accumulation over time (Fig. 5b). Internalized proteins are either recycled back to the cell surface for expression or ubiquitinated for protein degradation. We hypothesized that inhibition of proteasomal degradation could preserve MUC16/CA125 surface expression and potentially rescue the efficacy of BITES in HX cells. To evaluate this possibility, we treated OVCAR3 cells exposed to HX conditions with various proteosome inhibitors; MG132, bortezomib, ixazomib and carfilzomib. We determined the IC_50_ for each of these drugs to ensure that we were not using cytotoxic concentrations (Fig. 5c, Supplementary Fig. 6a). Only MG132 treatment increased CA125 expression under HX conditions (Fig. 5c, Supplementary Fig. 6b). Full western blot images are shown in Supplementary Fig. 11. Inhibition of HIF-1α did not improve CA125 expression under HX conditions (Supplementary Fig. 6c). Surprisingly, treatment with MG132 did not significantly improve the efficacy of BITEs against HX cells (Fig. 5d). This data suggests that MUC16/CA125 expression was necessary, but not sufficient for effective BITE-medicated cytolysis under HX conditions.

### Effect of hypoxia-induced VEGF on MUC16-BITE efficacy

**Fig. 6:**
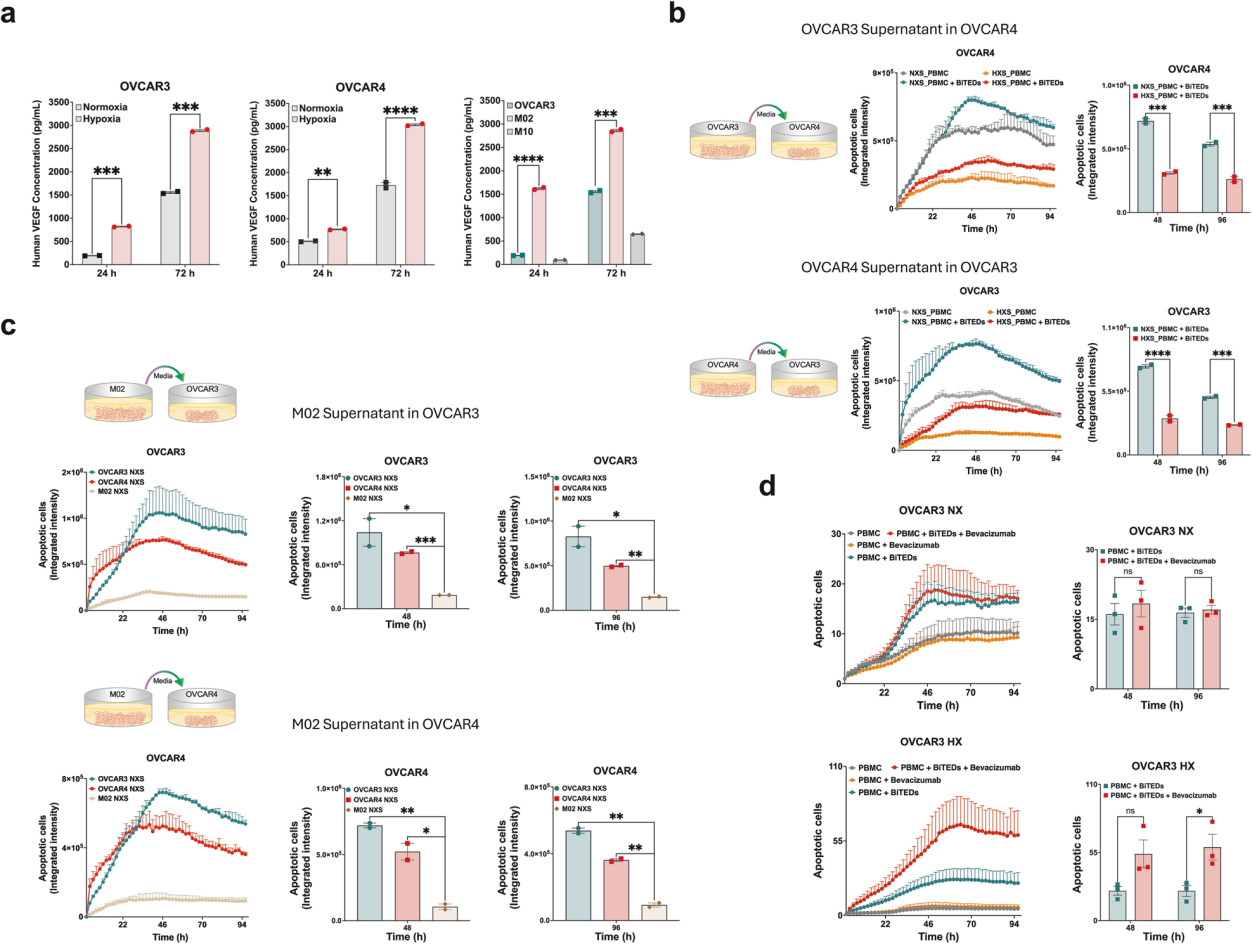
Evaluation of VEGF secretion under HX conditions. **a** Evaluation of VEGF secretion by ELISA in OVCAR3, OVCAR4, M02 and M04 cells cultured for the indicated timepoints. **b** Evaluation of MUC16-BITED cytotoxicity in OVCAR4 cells in the presence of OVCAR3 HX conditioned media (top row) and OVCAR3 cells in the presence of OVCAR4 HX conditioned media (bottom row). **c** Evaluation of MUC16-BITED cytotoxicity in OVCAR3 and OVCAR4 cells in the presence of M02-conditioned media (M02 NXS). Conditioned media from OVCAR3 HX (NXS) and OVCAR4 (NXS) were used as controls. **d** Evaluation of MUC16-BITED cytotoxicity in OVCAR3 NX and HX cells with or without bevacizumab. Data are expressed as the means ± SEM from two independent measurements for (**a**), (**b**), and (**c**), and two independent measurements for (**d**). Differences between the groups were analyzed using the Two-way ANOVA. **p < 0.01; ***p < 0.001; ****p < 0.0001

To explain why restoration of CA125 expression on HX cells was still inadequate to promote cytotoxicity in response to BITEs, we looked for additional factors that could be present in HX conditions over NX conditions. Based on our prior work, we were aware of VEGF’s effect on bispecific engager-based cytotoxicity.^22^ We found that VEGF secretion was significantly increased in OVCAR3 and OVCAR4 cells under HX conditions compared to NX as early as 24 h as measured by ELISA (Fig. 6a). This effect was further pronounced at 72 h under these conditions. In patient tumor samples M02 and M10, VEGF secretion was similarly increased at 24 h and 72 h (Fig. 6a). To decrease the possibility that our results could be attributed to cell line specific findings, we performed conditioned media transfer experiments. Cell-free conditioned media from NX or HX cells were transferred to target HX or NX cells and exposed to BITEs to see if the conditioned media influenced BITE-mediated cytotoxicity. Transfer of conditioned media from OVCAR3 HX cells to OVCAR4 NX cells led to decreased cytotoxicity in OVCAR4 NX cells at 48 h (0.7 × 10^6^ vs. 0.3 × 10^6^, *p* < 0.001) and 96 h (0.5 × 10^6^ vs. 0.3 × 10^6^, *p* < 0.001) (Fig. 6b). Transfer of conditioned media from OVCAR4 HX cells to OVCAR3 NX cells also led to decreased cytotoxicity in the target OVCAR3 NX cells at 48 h (0.8 × 10^6^ vs. 0.3 × 10^6^, *p* < 0.0001) and 96 h (0.5 × 10^6^ vs. 0.3 × 10^6^, *p* < 0.001) (Fig. 6b). Since we found increased VEGF in media collected from M02 cells (M02 NXS), we treated OVCAR3 and OVCAR4 cells with BITEs in the presence of NX-conditioned media (OVCAR3/4 NSX) or M02-conditioned media (M02 NXS) (Fig. 6c). There was decreased cytotoxicity in OVCA3 cells treated in the presence of M02 NXS compared to OVCAR3 NSX at 48 h (0.2 × 10^6^ vs. 1.0 × 10^6^, *p* < 0.05) and 96 h (0.1 × 10^6^ vs. 0.8 × 10^6^, *p* < 0.05) and OVCAR4 NSX at 48 h (0.2 × 10^6^ vs. 0.8 × 10^6^, *p* < 0.0001) and 96 h (0.1 × 10^6^ vs. 0.5 × 10^6^, *p* < 0.001). In OVCAR4 cells, we also found decreased cytotoxicity in the presence of M02 NXS compared to OVCAR3 NSX (0.1 × 10^6^ vs. 0.7 × 10^6^, *p* < 0.01) and 96 h (0.1 × 10^6^ vs. 0.5 × 10^6^, *p* < 0.01) and OVCAR4 NSX at 48 h (0.1 × 10^6^ vs. 0.5 × 10^6^, *p* < 0.05) and 96 h (0.1 × 10^6^ vs. 0.4 × 10^6^, *p* < 0.01). This data shows that a secreted component in the media, in this case VEGF, mediates resistance to BITE cytotoxicity regardless of tumor type or MUC16/CA125 expression. To directly test this, we treated OVCAR3 NX and HX cells with the VEGF-A blocking antibody bevacizumab and found that bevacizumab rescued BITE efficacy in HX cells at 48 (54 vs. 24, ns) and 96 h (59 vs. 24 × 10^6^, *p* < 0.05), but not in NX cells (Fig. 6d). Addition of bevacizumab did not improve cytotoxicity in M02 cells (Supplementary Fig. 7a).

To isolate the contribution of MUC16/CA125 expression and VEGF inhibition to BITE inhibition, we used SKOV3-MUC16^ecto^ cells.^22^ These cells do not downregulate MUC16/CA125 under HX conditions (Supplementary Fig. 7b). Treatment of SKOV3-MUC16^ecto^ HX cells with MUC16 BITEs led to significantly decreased toxicity compared to NX cells at 48 h and 96 h (Supplementary Fig. 7c), underscoring detrimental effect of VEGF on BITE activity. Inhibition with anti-PD1 antibodies did not rescue BITE activity in OVCAR3 HX cells (Supplementary Fig. 7d). Finally, we knocked out VEGF in OVCAR3 cells using CRISPR/Cas9 and confirmed decreased secretion of VEGF in these cells (OVCAR3^VEGFA KO^) compared to wild type OVCAR3 cells (Supplementary Fig. 8a). OVCAR3^VEGFA KO^ cells also downregulated CA125 under HX conditions (Supplementary Fig. 8b). VEGF knockout led to an increase in BITE-mediated cytotoxicity under NX conditions at 96 h, but not 48 h (Supplementary Fig. 8c); cytotoxicity was observed at 48 h and 96 h under HX conditions (Supplementary Fig. 8d).

### T-cell suppression by VEGF and hypoxia

**Fig. 7:**
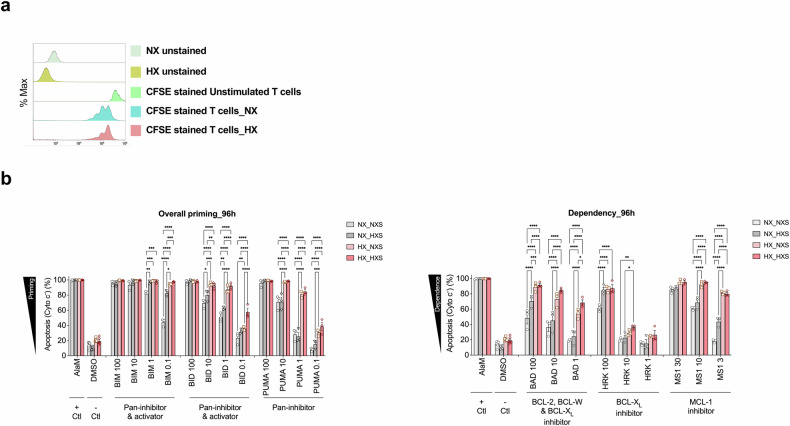
T-cell suppression by VEGF and hypoxia. **a** T-cell proliferation evaluated by CFSE labeling of T-cells stimulated in the context of NX or HX conditioned media. **b** BH3 profiling assay evaluating activated T-cells cultured under normoxic conditions (NX) with normoxic supernatant (NXS) or hypoxic supernatant (HXS) and activated T-cells cultured under hypoxic conditions (HX) with normoxic supernatant (NXS) or hypoxic supernatant (HXS) and treated with indicated peptides for 60 min prior to fixation and cytochrome c detection. Data are expressed as the means ± SEM from two independent measurements. Differences between the groups were analyzed using the Two-way ANOVA. **p < 0.01; ***p < 0.001; ****p < 0.0001

To evaluate the mechanisms of T-cell suppression by VEGF and hypoxia, we cultured activated T-cells in the presence of conditioned media derived from NX or HX OVCAR3 cells. We found decreased T-cell proliferation under HX conditions (Fig. 7a), providing a potential explanation for the suppressed cytotoxicity observed in presence of serum derived from M02 and M10 in Fig. 3b, c. To evaluate this further, we conduced BH3 profiling^30^ to evaluate apoptotic priming and T-cell dependency on pro-survival proteins in the presence of normoxic conditioned media (NXS) and hypoxic conditioned media (HXS) under both NX and HX conditions. After 96 h of culture, we found that T cells were highly primed for apoptosis under all experimental conditions, as demonstrated by robust cytochrome c release in response to pro-apoptotic BIM and BID BH3 peptides, even at low concentrations such as 1 µM BIM (Fig. 7b). T-cells cultured in hypoxic supernatant medium under normoxic conditions showed increased apoptotic priming and enhanced dependence on BCL-XL and MCL-1, indicating elevated mitochondrial priming driven by VEGF-rich conditions (Fig. 7b). T-cells cultured under normoxic conditions exhibited dependency on BCL-XL and MCL-1 for survival, as indicated by cytochrome c release in response to BAD (which inhibits both BCL-XL and BCL-2) and HRK (which selectively inhibits BCL-XL) (Fig. 7c). T-cells cultured in normoxic supernatant medium under hypoxic conditions exhibited further increased apoptotic priming and broader dependency on BCL-XL, BCL-2, and MCL-1, reflecting apoptotic sensitization under hypoxic stress. T-cells cultured in hypoxic supernatant medium under hypoxic conditions demonstrated the highest level of apoptotic priming and strongest dependency on BCL-XL, BCL-2, and MCL-1, indicating maximal apoptotic sensitivity when both hypoxia and high VEGF are present.

## Discussion

In this report, we identify and validate novel mechanisms of resistance to bispecific T-cell engager therapy in ovarian cancer. Using samples from patients with progressive disease on a clinical trial evaluating bispecific antibodies targeting MUC16, we found that target-antigen downregulation and VEGF secretion by the progressing lesions from the peritoneal compartment drove resistance to effector T-cell activity.

The only bispecific T-cell engagers currently approved for solid tumors are tebentafusp (CD3 x gp100) for uveal melanoma^15^ and tarlatamab (CD3 x DLL3) for small cell lung cancer.^16^ Hence, data regarding potential mechanisms of resistance in solid tumors are not readily available. In hematologic malignancies such as multiple myeloma, where BITEs such as teclistamab (CD3 x BCMA), elranatamab (CD3 x BCMA) and talquetamab (CD3 x GPRC5D) are approved, potential mechanisms of resistance have been identified. For instance, loss of GPRC5D was a common finding in multiple myeloma resistance to therapy,^31,32^ whereas loss of BCMA was a less frequent event.^33^ This is hypothesized to be due to the relative importance of BCMA to the survival of the myeloma cells compared to GPRC5D. In ovarian cancer, MUC16/CA125 overexpression drives oncogenic transformation^34^ and is associated with poor oncologic outcomes in patients.^18,35,36^ While MUC16/CA125 is also expressed on healthy ocular surface epithelia, pleural and peritoneal linings, and gynecological tissue,^37^ its expression has been shown to be entirely dispensable in mice.^38^ In this context, MUC16/CA125 downregulation in ovarian cancer as a mechanism of resistance to BITEs was unexpected. To our knowledge, this is the first report describing this phenomenon in a solid tumor malignancy. Intriguingly, all patients on the study with progressive disease, including M02 and M10 had rising serum CA125 despite low expression on the tumor. There are potential explanations for this finding. There is extensive intratumoral and metastatic site heterogeneity in ovarian cancer.^39,40,41^ Our tumor samples were limited to the ascites and pleural effusions. Disease from other metastatic sites may have contributed to an overall rise in the measured shed component of CA125 while progressing lesions might have decreased MUC16/CA125 expression. MUC16/CA125 shedding has also been shown to increase from benign tissues during inflammatory conditions.^42^ Since BITE therapy induces an inflammatory state with activation of cytotoxic T-cells and NK cells leading to secretion of cytokines such as TNF-α and IFN-γ, CA125 upregulation in non-malignant tissue in response may also have contributed.^37^

Acute and chronic inflammation have been reported to play opposing roles in anti-tumor immunity. Acute inflammation is generally recognized to promote an anti-tumor immune response, while chronic inflammation has been shown to be detrimental (reviewed in ref. ^43^). Increased levels of cytokines such as IL8,^44^ IL18,^45^ and IL1^46^ in our sampled population of patients who pressed on therapy is concerning because these cytokines have been shown to be involved in chronic inflammation. This raises the concern that at some juncture, repeat activation of T-cells via weekly BITE administration might be detrimental to some patients. Reassuringly, in our report, this was not a major driver of disease progression. However, investigations into the contribution of chronic T-cell activation and inflammation with BITE could yield additional mechanisms of resistance in non-peritoneal disease progression in ovarian cancer or other solid tumor malignancies.

In both patient samples, loss of MUC16/CA125 was concurrently present with an epithelial-to-mesenchymal transition. If MUC16/CA125 downregulation is therapy-related and this leads to a more aggressive disease phenotype, this would be a concerning finding. Comamala et al.^47^ showed that single-chain antibody mediated knockdown of MUC16/CA125 in OVCAR3 tumor cells led to an EMT phenotype. This transformation was linked to the signaling, morphological, and phenotypic changes associated with aggressive transition. We replicated this finding using CRISPR/Cas9 knockout of MUC16/CA125 in two different ovarian cancer cell lines and showed the same EMT transition associated with increased invasiveness, migration, and loss of sensitivity to BITE-mediated cytotoxicity.

The detrimental role of hypoxia in response to immunotherapy is well-described.^48^ In particular, hypoxia has been shown to accelerate MHC-I downregulation in an HIF-dependent manner.^49^ We found that the hypoxic peritoneal tumor microenvironment provides the ideal circumstance for MUC16/CA125 downregulation. We were again able to show this in two different ovarian cancer cell lines. We also looked at several different members of the mucin family under hypoxic conditions and found that mucin downregulation was not a universal phenomenon. Reassuringly, the presence of anti-MUC16 antibodies did not accelerate hypoxia-induced downregulation in our ovarian cancer models. Although inhibition of proteasomal degradation minimized downregulation of surface MUC16/CA125 expression, this did not fully restore sensitivity to BITE-medicated cytotoxicity. Inhibition of HIF-α did not restore MUC16/CA125 expression. This data suggests that antigen presentation is necessary, but not sufficient for optimal BITE activity. A limitation of our findings is that we did not sample other tumor sites such as nodal or liver metastasis to assess if this was a universal finding.

The finding that serum samples from patients who progressed on BITE therapy suppressed BITE cytotoxicity ex vivo, even though PBMCs from the same patients harbored no such deficits in ex vivo cytotoxicity experiments, suggests the contribution of suppressive elements that are not directly related to the fitness of the patients PBMCs or antigen expression. The role of hypoxia and VEGF upregulation in neovascularization, resistance to immunotherapy, and poor prognosis is well-described (reviewed in ref. ^50^). Our data showed that elevated levels of VEGF directly suppressed proliferation of activated T-cells and increased T-cell apoptotic priming in addition to suppressing BITE cytotoxic activity. Our conditioned media and patient serum sample transfer experiments showed that the presence of VEGF alone was sufficient to blunt the cytotoxic response to BITE therapy. Because VEGF is a soluble protein, it is conceivable that each patient’s progression might start in a hypoxic environment where MUC16/CA125 downregulation and EMT transition are initiated. These progressing cells then secrete increasing levels of VEGF into systemic circulation, rendering remote cancer cells in non-hypoxic conditions resistant to BITE therapy. Knocking out VEGF or blocking VEGF with bevacizumab, an approved monoclonal antibody therapy, significantly restored sensitivity to BITE therapy. In a previous report, we showed that the combination of MUC16-directed BITE therapy with bevacizumab was superior to BITE therapy alone or BITEs in combination with anti-PD-1 treatment in a mouse model of peritoneal HGSOC.^22^ In that report, we hypothesized that the reduction of ascites and its detrimental effect on effector T-cells were primarily responsible for the improved efficacy and prolonged survival. This current report provides mechanistic links between the hypoxic TME, MUC16/CA125 downregulation, VEGF upregulation, and resistance to BITEs.

Limitations of our study include tumor sampling primary from peritoneal ascites, which may not be representative of all metastatic sites. Furthermore, our tumor samples from M02 and M10 may not fully recapitulate the all the potential mechanisms of resistance to BITE therapy. Since emergence of ascites or malignant pleural effusions is a clinically significant event, which often signifies treatment failure, knowledge gained from this mechanism of progression has intrinsic value. Another limitation is that this study only included patients experiencing progression. It is not known if patients who are responding to therapy also have low MUC16/CA125 tumor expression or high levels of VEGF. This translational study was specifically intended to evaluate mechanisms involved in disease progression on immunotherapy. Further results on responders will be available once the trial completes accrual.

In conclusion, we propose that in a hypoxic tumor microenvironment, target-antigen downregulation, EMT transformation, and VEGF-secretion cooperatively mediate resistance to MUC16 BITE-medicated cytotoxicity in epithelial ovarian cancer cells. Treatment with bevacizumab ameliorates this suppressive effect and could be considered as a therapeutic approach to mitigating resistance to bispecific T-cell engagers.

## Materials and methods

### Patient and healthy donor PBMC and serum

Blood samples were collected prior to bispecific treatment initiation and at disease progression. Peripheral blood mononuclear cells (PBMCs) were isolated from patient blood samples collected in EDTA tubes. Blood was transferred to CPT tubes and processed to generate serum and PBMCs as previous described.^22^ Healthy donor T-cells were isolated from 50 ml donor whole blood (Research Blood Components, Watertown, MA). Peripheral blood mononuclear cells (PBMC) were separated using density gradient centrifugation with CPT tubes (BD Medical). The CPT tubes were centrifuged at 1700 × *g* for 20 min at 28 °C. The supernatant was carefully transferred to a 50 ml Falcon tube. The remaining cells were recovered by rinsing the CPT tube with 5 ml of PBS, which was subsequently combined with the initial supernatant. The pooled suspension was centrifuged at 1500 RPM for 5 min at 4 °C. The resulting pellet was resuspended in 1 mL of ACK lysis buffer and incubated for 5 min at room temperature, followed by neutralization with culture medium and an additional centrifugation step. The final cell pellet resuspended in RPMI growth medium, counted, and processed for either cryopreservation as PBMC.

### Ascitic tumor cells from patients

Ascitic fluid (500 mL to 1 L) was collected under sterile conditions from patients experiencing progression on BITE therapy. The fluid was transferred to 50 mL conical tubes and centrifuged at 400 × *g* for 10 min at room temperature to pellet cells. The supernatant was discarded, and the cell pellet was resuspended in ACK Lysing Buffer (Gibco, Thermo Fisher Scientific) to lyse red blood cells, following the manufacturer’s instructions. After incubation for 3–5 min, the cells were washed twice with sterile phosphate-buffered saline (PBS) and centrifuged at 400 × *g* for 5 min to remove residual lysing buffer.

For culture, 1 million viable cells were seeded onto 10 cm tissue culture–treated dishes (Corning Incorporated, NY, USA) in RPMI culture medium (Gibco) containing GlutaMAX™, supplemented with 10% heat-inactivated FBS, penicillin/streptomycin (100 U/mL and 100 μg/mL, respectively), and amphotericin B (Fungizone, InvivoGen, 2.5 μg/mL). Cultures were maintained at 37 °C in a humidified atmosphere of 5% CO₂.

Non-adherent cells were removed after 24–48 h by gentle media change. The adherent population, which typically adopted an elongated, mesenchymal morphology within 2–3 days, was expanded and fibroblast-depleted using differential trypsinization or negative selection as needed. Differential trypsinization was performed by adding trypsin to the adherent cells for 5–7 min and then neutralized by adding complete medium. Fibroblasts are more adherent than tumor cells and will remain on the plate after short trypsinization and neutralization, while epithelial ovarian cancer cells will have detached into the supernatant. Negative fibroblast exclusion was performed using the anti-fibroblast magnetic microbeads and separation column from Miltenyi Biotec (Cat # 130-050-601) according to the manufacturer’s instructions. Cells were passaged upon reaching ~80% confluency and maintained for downstream experiments, including screening assays.

### Immunohistochemical staining

Cells grown on glass slides (FALCON, #354104) were first washed twice in PBS, then fixed in cold acetone (–20 °C) for 10 min and rinsed three times in PBS. Slides were permeabilized in 0.3% Triton X-100 for 10 min, washed again, then endogenous peroxidase was quenched with 3% H₂O₂ before three PBS washes. Non-specific binding was blocked with 5% normal donkey serum for 1 h at RT. Primary antibodies (PAX8, GeneTex, GTX101583, 1:200 dilution; WT1, Cell Signaling Technology, #13580, 1:100 dilution) were applied overnight at 4 °C, followed by three PBS washes and a 1 h RT incubation with HRP-linked secondary antibody (Anti-rabbit IgG, Cell Signaling Technology, #7074, 1:500 dilution). Rabbit IgG was used as a control for primary antibodies (Vector Laboratories, #I-1000-5, 1:500 dilution). After PBS rinses, slides were developed in freshly prepared DAB solution and rinsed in DI water, counterstained with hematoxylin and Scott’s tap-water bluing, then dehydrated through graded ethanol (50–100%) and xylene and cover slipped in neutral balsam. Mounted slides were air-dried in a fume hood prior to microscopic examination (Nikon, ECLIPSE Ni-U).

### Cell lines and cytotoxicity

OVCAR3, OVCAR4, SKOV3, HEK293T, IMR-90, and H226 cells were purchased from the American Type Culture Collection (ATCC, Washington, USA). SKOV3 modified to express MUC16^ecto^ and the luciferase gene (SKOV3-MUC16^ecto^-Luc), and wild-type isogenic SKOV3-Luc cells have been described previously.^22^ M02 and M10 cells were isolated from patient ascites. All human ovarian cancer cell lines were maintained in RPMI (Gibco, Grand Island, NY, USA) supplemented with 10% heat-inactivated fetal bovine serum (FBS), 100 IU/ml penicillin and 100 mg/ml streptomycin (P/S), and 2 mM L-glutamine. HEK293T cells were cultured in GIBCO Dulbecco’s modified Eagle’s medium (DMEM) supplemented with 10% fetal bovine serum (FBS, Lanzhou Bailing) and 100 units/mL penicillin, 100 mg/mL streptomycin and 2 mM L-glutamine, incubated in a humidified 5% CO_2_ incubator at 37 °C. Cells were validated using karyotype analysis and routinely checked for mycoplasma contamination.

For BITE-variable killing assays, tumor cells and activated T-cells at varying effector:target (E:T) ratios in the presence of increasing concentrations of MUC16 BITEs for 48 h and subsequently mixed with luciferase assay reagent (Promega). Luminescence of the lysates was analyzed using a plate spectrophotometer. Specific cytolysis was calculated using the formula; % specific lysis = 100 × (sample lysis-spontaneous lysis)/(maximal lysis-spontaneous lysis).

For PBMC-variable killing assays, both SKOV3 (MUC16^ecto^)-Luc and OVCAR3-Luc were separately co-cultured with 5 µg/mL BITEs and either healthy donor PBMCs or patient-derived PBMCs for 48 h and subsequently mixed with luciferase assay reagent (Promega). Luminescence of the lysates was analyzed using a plate spectrophotometer. Specific cytolysis was calculated as above.

For serum-variable killing assays, tumor cells and Jurkat cells were co-cultured 1:1 with various concentrations of serum (0%, 10%, 50%, and 100%) from either healthy donors or patients in the presence of 5 µg/mL BITEs for 48 h. Assessment of cytolysis was performed as above.

### Cytokine measurement and ELISA

Cytokine detection was performed using the Human Cytokine & Chemokine Panel 1A, 34plex kit (Thermo Fisher Scientific, USA), and the Luminex IS100 system according to the manufacturer's protocol.

Human CA125/MUC16 ELISA kit and Human VEGF ELISA Kit (R&D Systems, MN, USA) were used according to the manufacturer’s protocol. 1 × 10^6^ cells of each type were cultured for 48 h and then underwent a media change. Twenty-four hours following media change, cell supernatant was collected for analysis. Cell numbers from the culture plate were then determined to allow for quantification of CA125 and VEGF secretion excretion per unit cells. Wavelengths of 450 nm and 540 nm (for plate refraction correction) were measured via SpectraMax iD3 Microplate Reader. CA125 and VEGF concentrations were determined by constructing a standard curve using the provided standards.

### Expression, production, and purification of MUC16^ecto^-BITEs

We have previously described process of MUC16^ecto^-BITE production in detail.^22^ Briefly, the E-ALPHA® human phage display library was used to screen for clones that specifically bind to MUC16 ectodomain. Positive phage clones were further characterized by FACS to identify clones with specific binding to MUC16^ecto^ and not mutant forms of MUC16^ecto^. Sequence-validated candidates with a hexahistidine (His) tag downstream of the BITED at the C-terminal end were cloned into CHO cells in serum-free media. Secreted MUC16^ecto^-BITEDs were purified using a HisTrap HP column and eluted into PBS for stock and single use aliquots.

### Hypoxia experiments

Hypoxic cell culture was carried out in a Panasonic MCO-5M hypoxia incubator. Nitrogen and carbon-dioxide cylinders were each connected to their designated inlets and the regulators adjusted to 20 psi (N₂) and 15 psi (CO₂), respectively. The chamber was then programmed to 37 °C, 5% CO₂ and 1% O₂ and allowed to equilibrate for 12–24 h, during which O₂, CO₂ and temperature were monitored continuously to confirm stability. For each experiment, the pre-equilibrated chamber was purged for at least 1 h (to re-establish 1% O₂) before seeding cells in standard culture medium. Cultures were then maintained at 37 °C under an atmosphere of 1% O₂, 5% CO₂ and 94% N₂ for the indicated timepoints, with gas concentrations continuously monitored throughout.

### FACS analyses

Flow cytometry analyses were performed using Gallios Flow Cytometer with Kaluza Software (Beckman Coulter). For cells that were to undergo intracellular staining, treatment for 2 h with GolgiStop and GolgiPlug (BD Biosciences) occurred prior to FACS preparation. For both tumor cells and PBMCs undergoing surface antibody staining, cells were pelleted and washed 3 times with FACS buffer (PBS + 2.5% FBS). Cells were then resuspended with the appropriate antibody, diluted in FACS buffer, and incubated at 4 °C for 30 min in the dark. The cells were subsequently washed 3 times with cold FACS buffer. Intracellular antibody staining was achieved by fixing and permeabilizing cells (Cytofix/Cytoperm, BD Biosciences), washing them 3 times with cold, diluted Perm/Wash buffer (BD Biosciences), resuspended with the appropriate antibody, diluted in Perm/Wash buffer, and incubated at 4 °C for 30 min in the dark. The cells were subsequently washed 3 times with cold Perm/Wash buffer and read.

Expression of tumor markers were determined with anti-CA125 (APC, R&D 986808) and anti-MUC16^ecto^ (APC conjugated anti-MUC16 antibody). Patient PBMCs were analyzed for type, activity, and exhaustion with anti-CD3, anti-CD4 (PE-Cy7, BD Biosciences RPA-T4), anti-CD8 (PE-Cy7, BD Biosciences HIT8α), anti-TIM3 (FITC, BioLegend F38-2E2), anti-LAG3 (APC, R&D polyclonal), anti-CD25 (FITC, BioLegend BC96), anti-FOXP3 (APC, Miltenyi Biotec REA1253), anti-TNF-α (Alexa Fluor 405, R&D 6401), and anti-IL2 (APC, BioLegend MQ1-17H12).

### Cell migration and invasion assays

Modified 24-well (8.0-μm pore size) chamber with and without matrix gel (Millipore) was inserted into a 24-well plate containing 500 μl complete medium. Then, 1 × 10^5^ cells in 200 μl basic medium containing 1% FBS were seeded into the top chamber and cultured for the indicated time. Later, the inner of the chamber was carefully scraped by a swab and stained with crystal violet for 30 min. The number of invasive cells was counted from the image taken with a microscope.

### Real-time cytotoxicity assay

Cell impedance cytotoxicity experiments were performed on a Live-Cell Imaging and Analysis instrument (Sartorius, Germany). Respective target cells (ovarian cancer) were seeded in 96-well flat and clear-bottom black plates (ThermoFisher Scientific) at 50,000 cells per well in a total volume of 200 μL per well. After allowing cells to adhere, PBMCs from healthy donors were added at an E:T ratio of 1:1. Treatment groups included the addition of BITEs (5 μg/mL), inhibitors, and Annexin V Red Dye (1:200 final dilution, Sartorius). Control wells contained tumor cells alone or untreated wells. Plates were placed in the Live-Cell Analysis System, and imaging was performed using a 20× objective with the phase contrast and red fluorescence channels. Scans were conducted every 3 h for 4 days. The software provided by the manufacturer was used to analyze the cell index and calculate specific lysis based on fluorescence signals.

### Western blotting

Tumor cells were collected and lysed with lysis buffer [50 mM Tris-HCl (pH 7.5), 150 mM NaCl, 1 mM EDTA, 1% Triton X-100, 20 mM DTT, and proteinase inhibitor cocktail]. The concentrations of proteins were measured by Protein Assay Dye, and 20 μg of total protein were loaded into Bis-Tris gels. Proteins were transferred to PVDF membranes. The membranes were blocked with 5% skim milk and 5% Western blocking buffer for 1 hour at room temperature before incubation with primary antibodies and secondary antibodies [anti-mouse IgG-HRP (at 1:2000 dilution), anti-rabbit IgG-HRP (at 1:2000 dilution)]. The immunoreactive bands were visualized using Immobilon Western HRP Substrate and photographed by ChemiDocTM Imaging System. The following antibodies were used: CA125-M11 (1:1000, Agilent Technologies), E-cadherin (1:1000, CST), N-cadherin (1:1000, CST), Vimentin (1:1000, CST), PAX8 (1:5000, GeneTex), Twist (1:500, GeneTex), WT1 (1:1000, CST), Mesothelin (1:1000, CST), EGFR (1:500, CST), Snail (1:500, GeneTex), β-Actin (1:2000, CST), HIF1α (1:1000, Proteintech), Anti-rabbit IgG HRP conjugate (1:5000, CST), and Anti-mouse IgG HRP conjugate (1:5000, CST).

### BH3 profiling

BH3 profiling was conducted via flow cytometry following previously established protocols.^30^ In brief, T cells were pelleted and resuspended in Mannitol Experimental Buffer 2 (MEB2), composed of 10 mM HEPES (pH 7.5), 150 mM mannitol, 150 mM KCl, 1 mM EGTA, 1 mM EDTA, 0.1% BSA, and 5 mM succinate. The cell suspension was then transferred into prepared plates with the indicated BH3 peptide and 0.001% digitonin. Alamethicin (25 μM) served as a positive control, while DMSO was used as a negative control. After 60 min of incubation at 28 °C, cells were fixed by adding 8% paraformaldehyde at a 1:4 dilution (2% final) for 15 min, followed by neutralization with N2 buffer (1.7 M Tris base, 1.25 M glycine, pH 9.1). Fixed cells were stained overnight at 4 °C in intracellular stain buffer (0.2% Tween20, 1% BSA) containing DAPI (1:1000, Abcam) and anti-Cytochrome c-Alexa Fluor 647 (1:2000, clone 6H2.B4, Biolegend). Cytochrome c release was quantified using an Attune NxT flow cytometer (Thermo Fisher Scientific).

### Statistical analysis

Prism 10.1.1 software (GraphPad Software, San Diego, CA, USA) was used for statistical analysis. Quantitative data were presented as mean ± SEM, and statistical analyses were performed as described in the legends. Student’s t-test was performed to assess whether significant differences existed between groups. Multiple comparisons were analyzed using the Two-way ANOVA, and group comparisons were performed using Šidák correction. Differences are considered statistically significant at *p < 0.05; **p < 0.01; ***p < 0.001; ****p < 0.0001; ns means no significance.

## Supplementary information

Supplemental material

## Data Availability

All data are available in the main text or the supplementary materials.
