# Supplementary material for: Hypoxia induced VEGF secretion promotes resistance to bispecific T-cell engagers
Source: Signal Transduct Target Ther. 2025 Dec 19;10:405. doi: 10.1038/s41392-025-02505-3 (PMC12715234; doi:10.1038/s41392-025-02505-3)
Supplement: Supplementary file 1 — Supplemental material [file 41392_2025_2505_MOESM1_ESM.docx]

Supplementary Materials for

Hypoxia induced VEGF secretion promotes resistance to bispecific T-cell engagers

**Authors:** Mengyao Xu^1#^, Syem K. Barakzai^2#^, Raj Kumar ^1^, Irva Veillard ^1^, Eugene Kim ^1^, Amy Bregar^2^, Eric Eisenhauer^2^, Richard Penson^3^, Sara Bouberhan^3^, Jennifer Filipi^1^, Tina Colella^1^, Tim Bond^1^, Caroline Clark^1^, Lawrence H. Lin^4^ , Jinpeng Ruan^5^, Cheng Wang^5^, Xingping Qin^6^, Kristopher Sarosiek^6^, Bo Rueda^2,3^, Cesar Castro^3^, David R. Spriggs^3^, Oladapo O. Yeku ^3*^.

Correspondence to: Oyeku@mgh.harvard.edu

#: These authors contributed equally

**This PDF file includes:**

Materials and Methods

Patient Narratives:

Figures. S1 to S11

Table S1

Table S2

Materials and Methods

*Antibody Internalization Assay*

Cells were seeded in a 96-well plate (7,000 cells/well) and incubated overnight. The next day, test antibodies (h4H11, mVK8) were labeled with FabFluor-pH Red (Sartorius) at a 1:3 molar ratio and incubated at 37°C for 15 minutes in the dark. Labeled antibodies (4 µg/mL) were added to cells (50 µL/well). Live-cell imaging was performed every 30 minutes for 48 hours using the Live-Cell Analysis System (Sartorius), and internalization was analyzed using integrated software.

*Apoptosis Assay*

Apoptosis was assessed using the FITC Annexin V Apoptosis Detection Kit with 7-AAD (BioLegend). Cells were washed with cold buffer, resuspended in Annexin V Binding Buffer, and stained with FITC-Annexin V and 7-AAD. After 15 minutes of incubation at room temperature in the dark, samples were analyzed by flow cytometry to distinguish live, early apoptotic, and late apoptotic/necrotic cells based on Annexin V and 7-AAD staining.

*CRISPR/Cas9-mediated gene knockout (KO)*

MUC16-KO and VEGF-KO cell lines were generated using the CRISPR/Cas9 system. Oligonucleotides were cloned into the LentiCRISPRv2 vector. The vector was digested with BsmBI and ligated with the annealed sgRNA oligos. Constructs were confirmed by Sanger sequencing and packaged into lentiviral particles by co-transfection with psPAX2 and pVSV.G plasmids in HEK293T cells for lentivirus production. The oligonucleotide pair used was as follows: human MUC16 (F: 5′-CACC GAGG AGGA CATG CGGC ACCC-3′ and R: 5′-AAAC GGGT GCCG CATG TCCT CCTC-3′), human VEGF (F: 5′-CACCG ATGCG GATCA AACCT CACCA-3′ and R: 5′-AAAC TGGT GAGG TTTG ATCC GCAT C-3′). Knockout efficiency was verified by Western blotting, Flow cytometry and ELISA.

*Fibroblast Exclusion*

Patient ascites tumor cells were incubated with D7-FIB containing anti-fibroblast microbeads (Miltenyi Biotec, North Rhine-Westphalia, Germany). The cells were then washed with a PBS buffer containing 0.5% bovine serum albumin and 2mM EDTA. The cell suspension was passed through a magnetic separation column (Miltenyi Biotec). The magnetically retained cells were then removed from the column by removal from the magnetic field.

*T-Cell Proliferation Analysis*

T-cell proliferation was assessed using Carboxyfluorescein Succinimidyl Ester (CFSE) staining and flow cytometry. PBMCs were isolated from whole blood by density gradient centrifugation using Ficoll-Paque™ Plus (GE Healthcare, USA). Cells were washed twice with DPBS (Gibco, Thermo Fisher Scientific, USA) and resuspended at 1×10^6^ cells/mL. CFSE (Invitrogen, Thermo Fisher Scientific, USA) stock solution was prepared by dissolving in DMSO (Sigma-Aldrich, USA) and diluted in PBS (Gibco, Thermo Fisher Scientific, USA) to a final concentration of 5 µM. Cells were incubated with CFSE at 37°C for 20 minutes, followed by quenching with T Cell Expansion SFM (Gibco, Thermo Fisher Scientific, USA). Excess dye was removed by washing and centrifugation. CFSE-stained T cells were cultured in a regular oxygen-level incubator under several conditions: unstained NX cells, unstained HX cells, CFSE-stained unstimulated T cells, CFSE-stained T cells co-cultured with NX cells, and CFSE-stained T cells co-cultured with HX cells. All cultures were maintained in 6-well plates (Corning, USA). After the incubation period, cells were harvested and analyzed using flow cytometry (Beckman Coulter, CA, USA). Fluorescence intensity shifts in CFSE-labeled cells were used to assess cell division and proliferation. Data were analyzed using FlowJo software (BD Biosciences, USA) to quantify T-cell proliferation across experimental conditions.

*MUC16 Stability Assay under Hypoxic Conditions*

Cells were cultured in a hypoxia chamber (1% O₂) for 72 hours. After 72 hours, hypoxic cells were treated with either the proteasome inhibitor MG132 (CST) at a final concentration of 5 μM or the HIF-1α inhibitor YC-1 (Selleck) at 20 μM for an additional 12 hours prior to collection. Following treatment, cells were collected separately for cytotoxicity assay and Western blot analysis. Tumor cells were lysed using RIPA buffer (Thermo Fisher Scientific, USA) supplemented with protease and phosphatase inhibitors. Protein lysates were quantified using the BCA protein assay kit (Thermo Fisher Scientific, USA) and subjected to SDS–PAGE, followed by Western blotting.

*Quantitative Real-time PCR*

RNA was extracted using TRIzol reagent (Thermo Fisher Scientific) following the manufacturer’s protocol. Complementary DNA (cDNA) was synthesized from RNA using the iScript cDNA Synthesis Kit (Bio-Rad). Reactions were performed in a thermal cycler with the following conditions: priming at 25°C for 5 minutes, reverse transcription at 46°C for 20 minutes, and inactivation at 95°C for 1 minute. Synthesized cDNA was diluted 5-fold before use. qPCR reactions were prepared in a 20 μL volume containing iTaq Universal SYBR Green Supermix (Bio-Rad), forward and reverse primers (final concentration 300–500 nM), nuclease-free water, and 4 μL of diluted cDNA template. Reactions were run in a 96-well plate (VWR® PCR Plates) on a thermal cycler using the following cycling conditions: initial denaturation at 95°C for 30 s, followed by 39 cycles of denaturation at 95°C for 10 s and annealing/extension at 60°C for 30 s. A melt curve analysis was performed to ensure specificity of the amplified products. The primer sequences were listed in Supplementary Table S2.

**Patient Narratives:**

**M02** - The patient was diagnosed with stage IIIC high grade serous ovarian cancer in 2019 and underwent primary cytoreductive surgery followed by six cycles of platinum-based chemotherapy, completed in April 2020. She remained without evidence of disease until December 2020, when she experienced a recurrence and received six additional cycles of platinum-based chemotherapy, followed by maintenance therapy with a PARP inhibitor. Disease progression was noted in August 2021, at which point she initiated treatment with paclitaxel and bevacizumab, continuing until further progression in March 2022. She subsequently enrolled in a clinical trial MUC16 BITE therapy, which she received until May 2022. Plans for additional platinum-based chemotherapy were made; however, she elected for hospice care and died in August 2022.

**M10** - The patient was diagnosed in 2017 with stage IIIC high grade serous ovarian cancer and underwent optimal primary cytoreductive surgery followed by adjuvant intraperitoneal and intravenous cisplatin and paclitaxel, which she completed in April 2018. She experienced a recurrence in March 2019 and received six cycles of platinum-based chemotherapy, followed by maintenance therapy with a PARP inhibitor. In December 2020, she had disease progression and was treated with carboplatin, gemcitabine, and bevacizumab until March 2021, after which she continued on bevacizumab maintenance. Upon progression in July 2021, she received paclitaxel and bevacizumab, followed by pegylated liposomal doxorubicin and bevacizumab upon further progression in January 2022. In November 2022, she was enrolled on a clinical including MUC16 BiTEs. She remained on study until disease progression in January 2023, at which time she transitioned to hospice care. She died later that month.

Figure. S1.


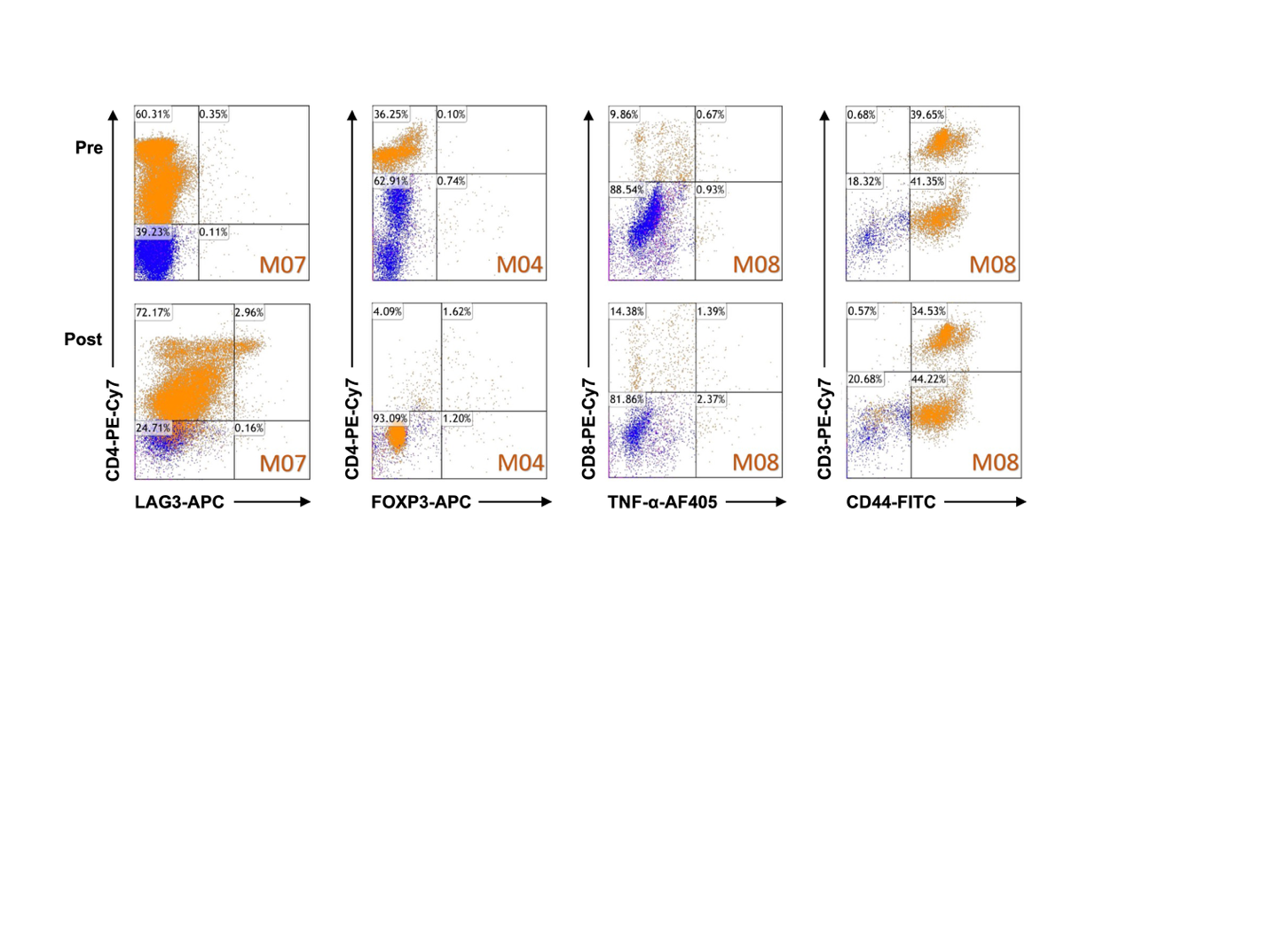


**Supplementary Figure 1: Flow Cytometry Gating Strategy**

Representative flow dot plots of patient PBMCs assessing exhaustion, Treg, Activated phenotype, and Memory phenotype before and after treatment.

Figure. S2.


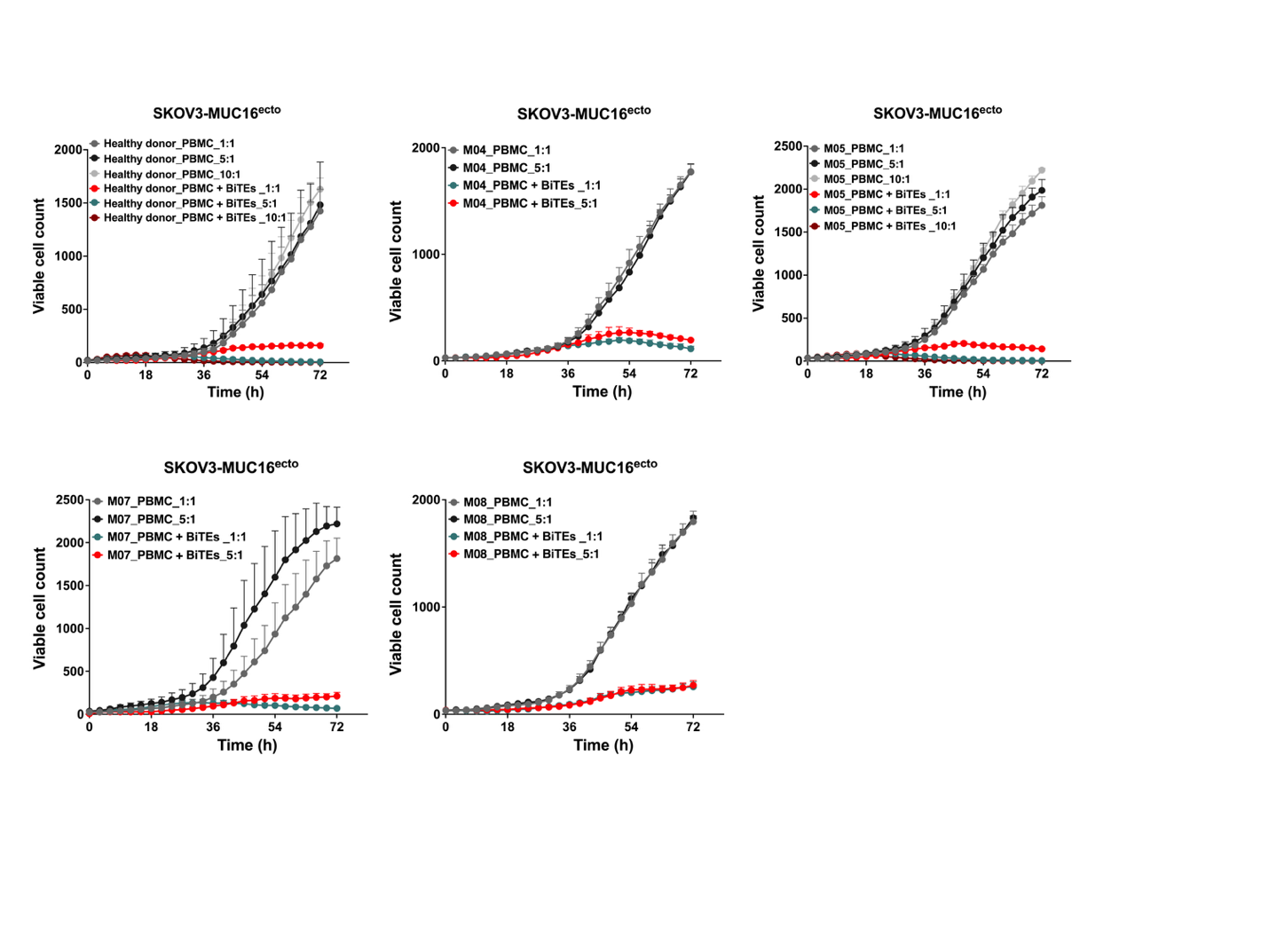


**Supplementary Figure 2: Patient PMBC cytotoxicity assay**

Co-culture of MUC16^ecto^ -BITEs and healthy donor PBMC, M04, M05, M07 and M08 PBMC with SKOV3-MUC16^ecto^ at the indicated E:T ratios.

Figure. S3.


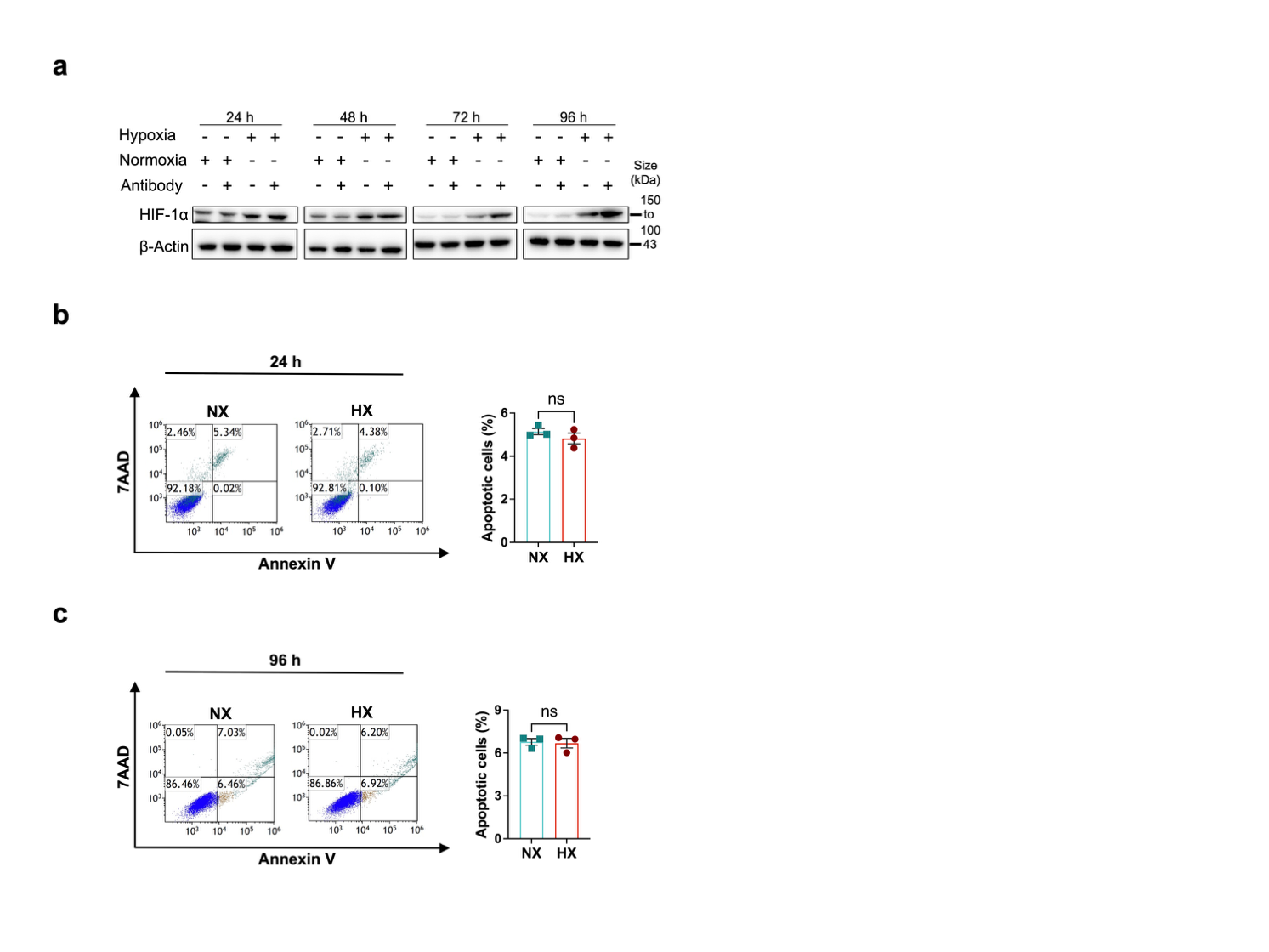


**Supplementary Figure 3: HIF-1α and apoptosis under hypoxic conditions**

(**a**). OVCAR3 cells cultured under NX and HX and subjected to Western blot analysis for HIF-1α. (**b**). Evaluation of apoptosis in OVCAR3 NX and HX via 7AAD and Annexin V staining and flow cytometry. ns, not significant.

Figure. S4.


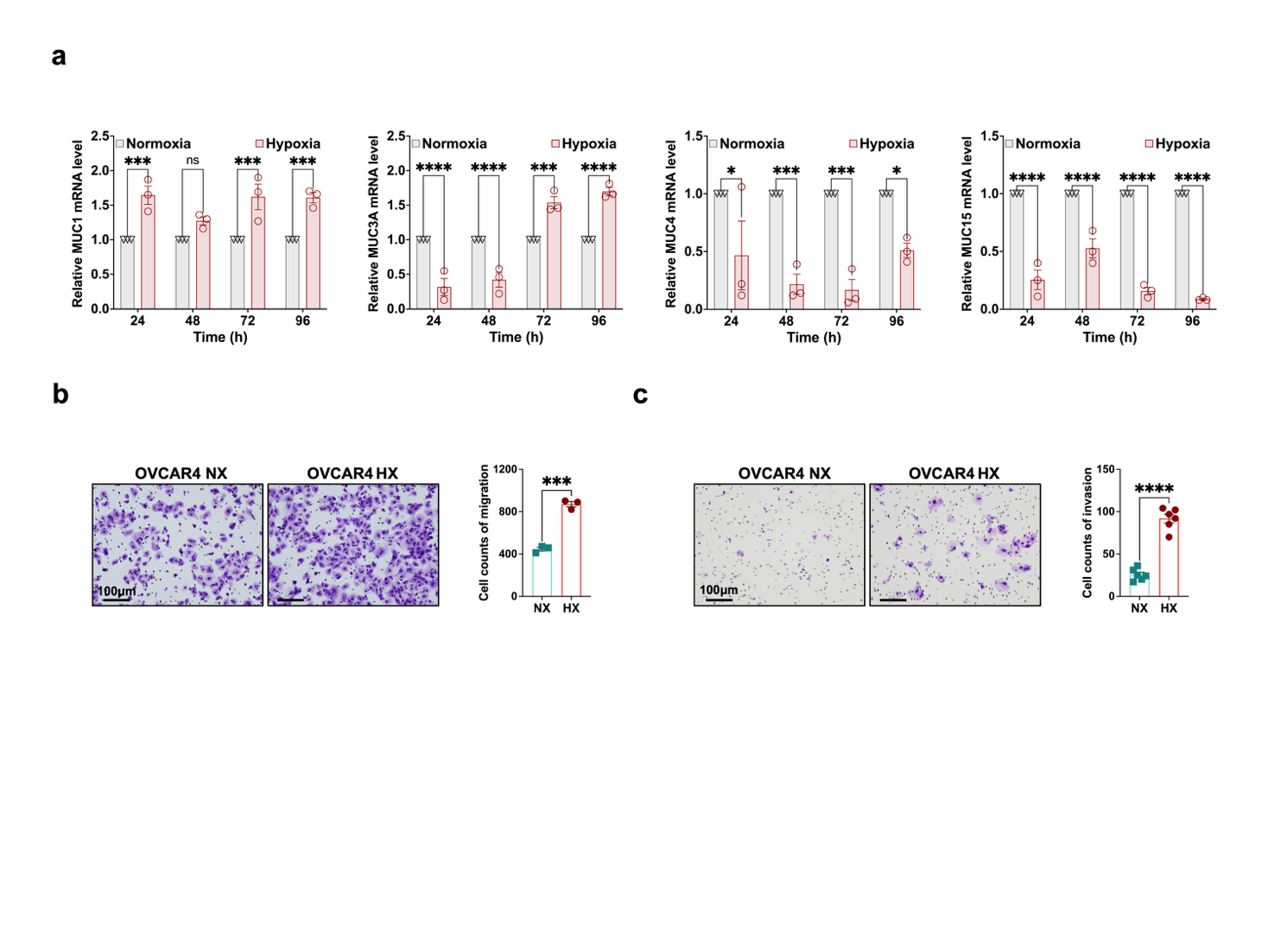


**Supplementary Figure 4: mRNA expression of mucins under hypoxic conditions and OVCAR4 invasion and migration**

(**a**). mRNA expression of Muc1, Muc3A, Muc4, and Muc15 in OVCAR3 cells cultured under NX and HX conditions for the indicated timepoints. All data are expressed as the means ± SEM from three independent measurements, and the differences between the groups were analyzed using the Two-way ANOVA. ns, not significant; *p<0.05; **p<0.01; ***p<0.001; ****p<0.0001. (**b**) OVCAR4 NX and HX cells evaluated for migration. (**c**) OVCAR4 NX and HX cells evaluated for invasion. All data are expressed as the means ± SEM from three independent measurements, and the differences between the groups were analyzed using the student’s t-test. ns, not significant; *p<0.05; **p<0.01; ***p<0.001; ****p<0.0001.

Figure. S5.


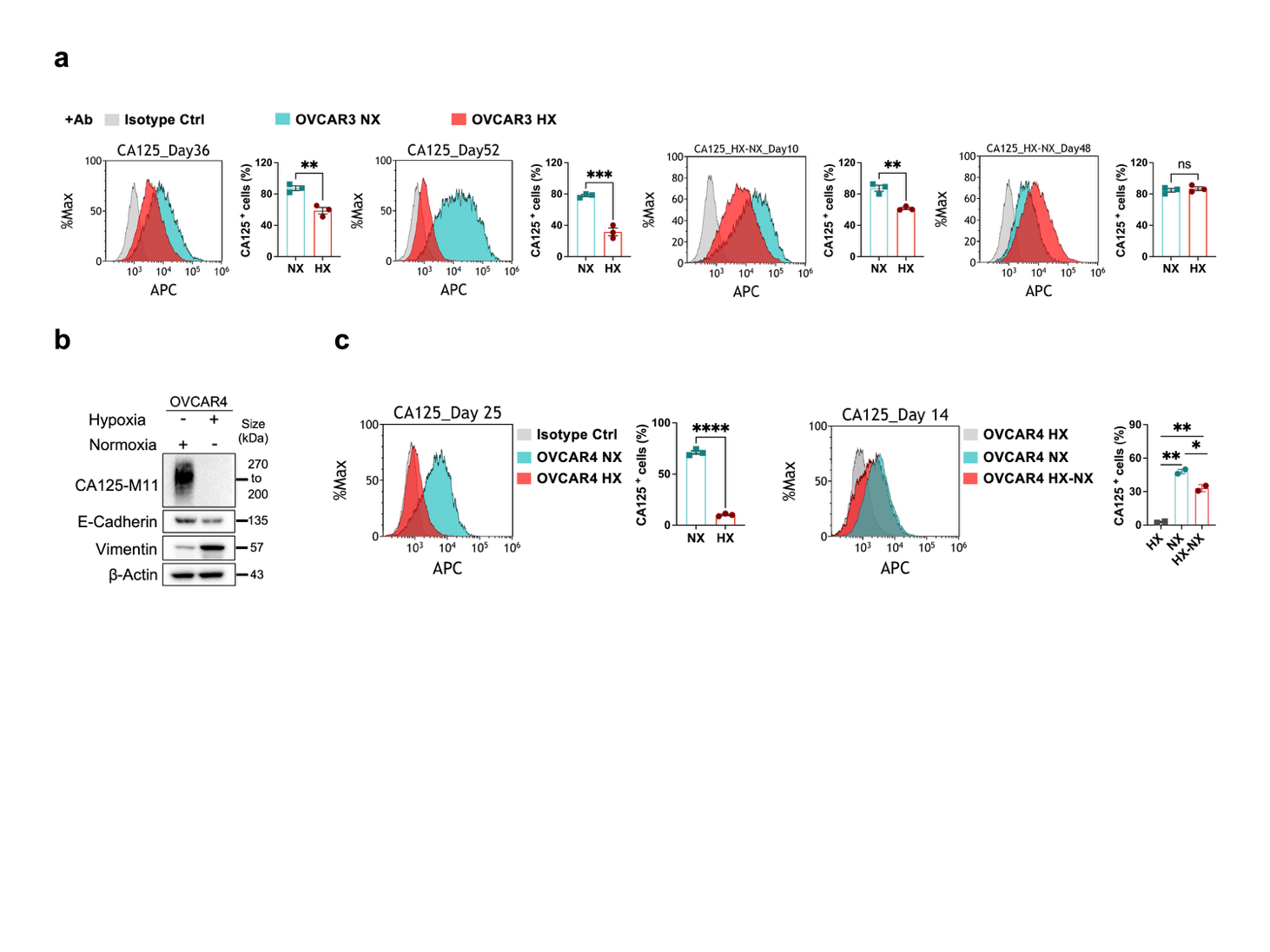


**Supplementary Figure 5: Expression of CA125 cultured under NX or HX conditions in the presence of anti-MUC16 antibody and expression of CA125 on OVCAR4 cells**

(**a**). CA125 expression by flow cytometry on OVCAR3 cells cultured in NX or HX in the presence of anti-MUC16^ecto^ antibodies for the indicated timepoints followed by transfer of the HX cells to NX culture (HX – NX) conditions for 10 days or 48 days. (**b**). Western blot analysis of OVCAR4 cells for CA125 expression and EMT markers (**c**). OVCAR4 cells cultured under NX or HX conditions, followed by HX –NX conditions for 14 days and 25 days. All data are expressed as the means ± SEM from two independent experiments for panel a, and three independent measurements for panel c at Day 25 and two independent measurements at Day 14, and the differences between the groups were analyzed using the student’s t-test. ns, not significant; *p<0.05; **p<0.01; ***p<0.001; ****p<0.0001.

Figure. S6.


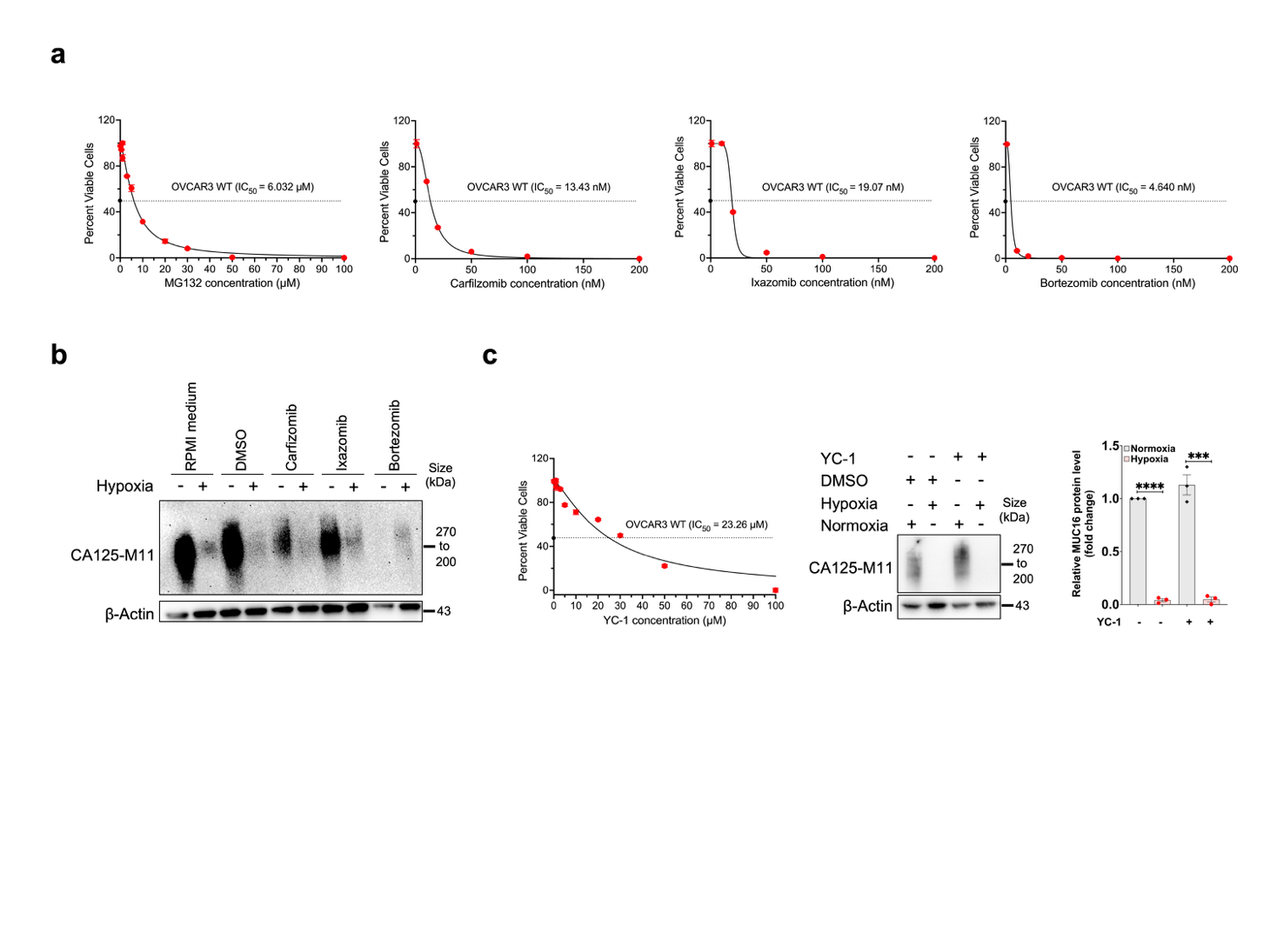


**Supplementary Figure 6: Evaluation of proteosome inhibitors on CA125 expression**

(**a**). Evaluation of IC_50_ concentrations for MG132, carfilzomib, ixazomib and bortezomib in OVCAR3 cells. (**b**). Western blot analysis of OVCAR3 cells treated with the indicated proteosome inhibitors and evaluated for CA125 expression (**c**). Evaluation of the IC_50_ of YC-1 in OVCAR3 cells (left panel) and western blot analysis for CA125 expression. Data are expressed as the means ± SEM from three independent measurements, and the differences between the groups were analyzed using the student’s t-test. ***p<0.001; ****p<0.0001.

Figure. S7.

**
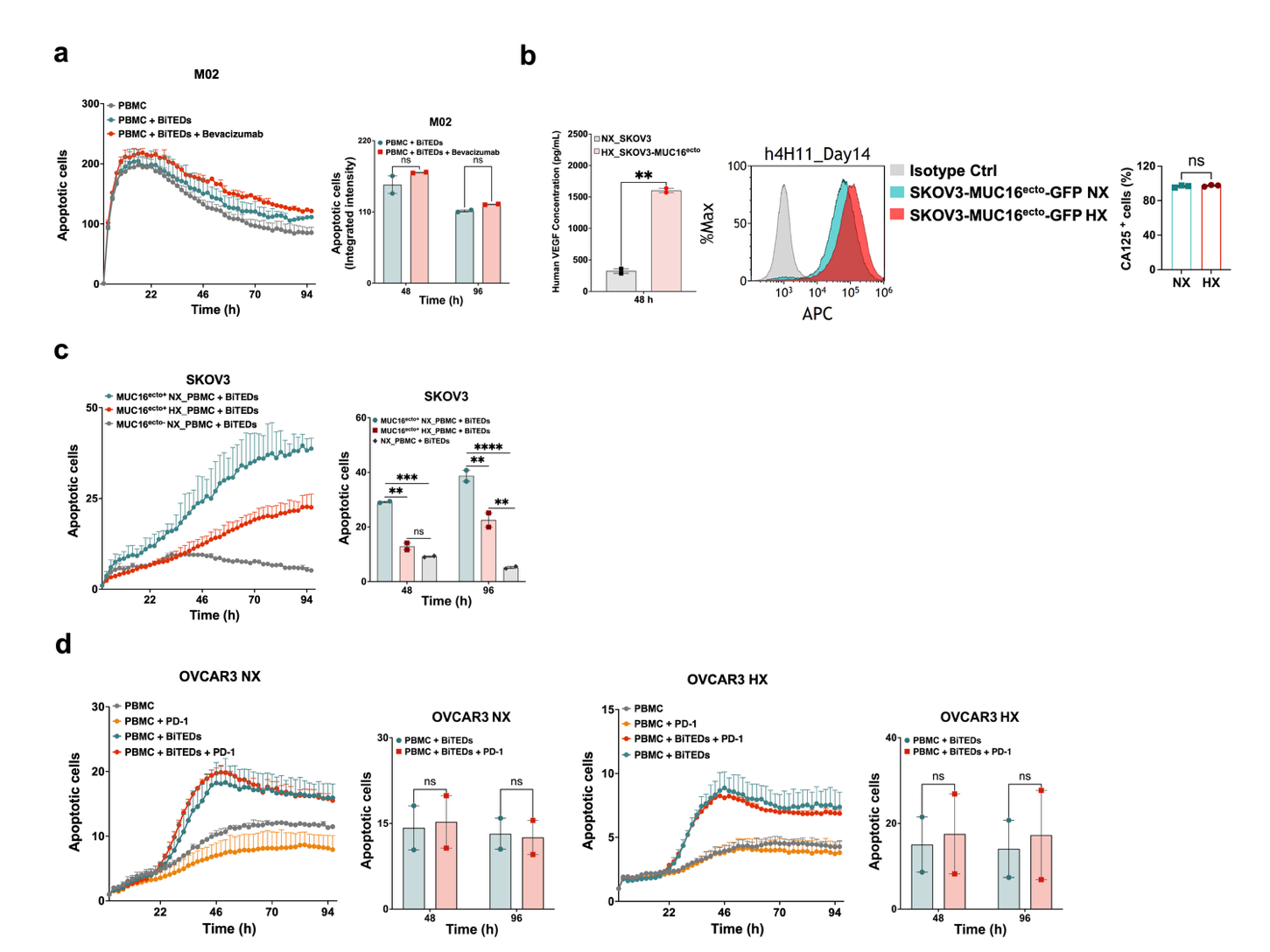
**

**Supplementary Figure 7: Evaluation of the effect of angiogenic blockade and immune checkpoint inhibition**

(**a**). Evaluation of MUC16-BITED cytotoxicity in M02 cells in the presence of bevacizumab. (**b**). CA125 surface expression on SKOV3-MUC16^ecto^ cultured under NX or HX conditions. (**c**). Evaluation of MUC16-BITED cytotoxicity in SKOV3-MUC16^ecto^ cultured under NX or HX conditions. (**d**). Evaluation of MUC16-BITED cytotoxicity in OVCAR3 NX and HX with or without anti-PD-1 immune checkpoint inhibitors. All data are expressed as the means ± SEM from two independent measurements for panel a, c, d, and three independent measurements for panel b, and the differences between the groups were analyzed using the student’s t-test. ns, not significant; **p<0.01; ***p<0.001; ****p<0.0001.

Figure. S8.


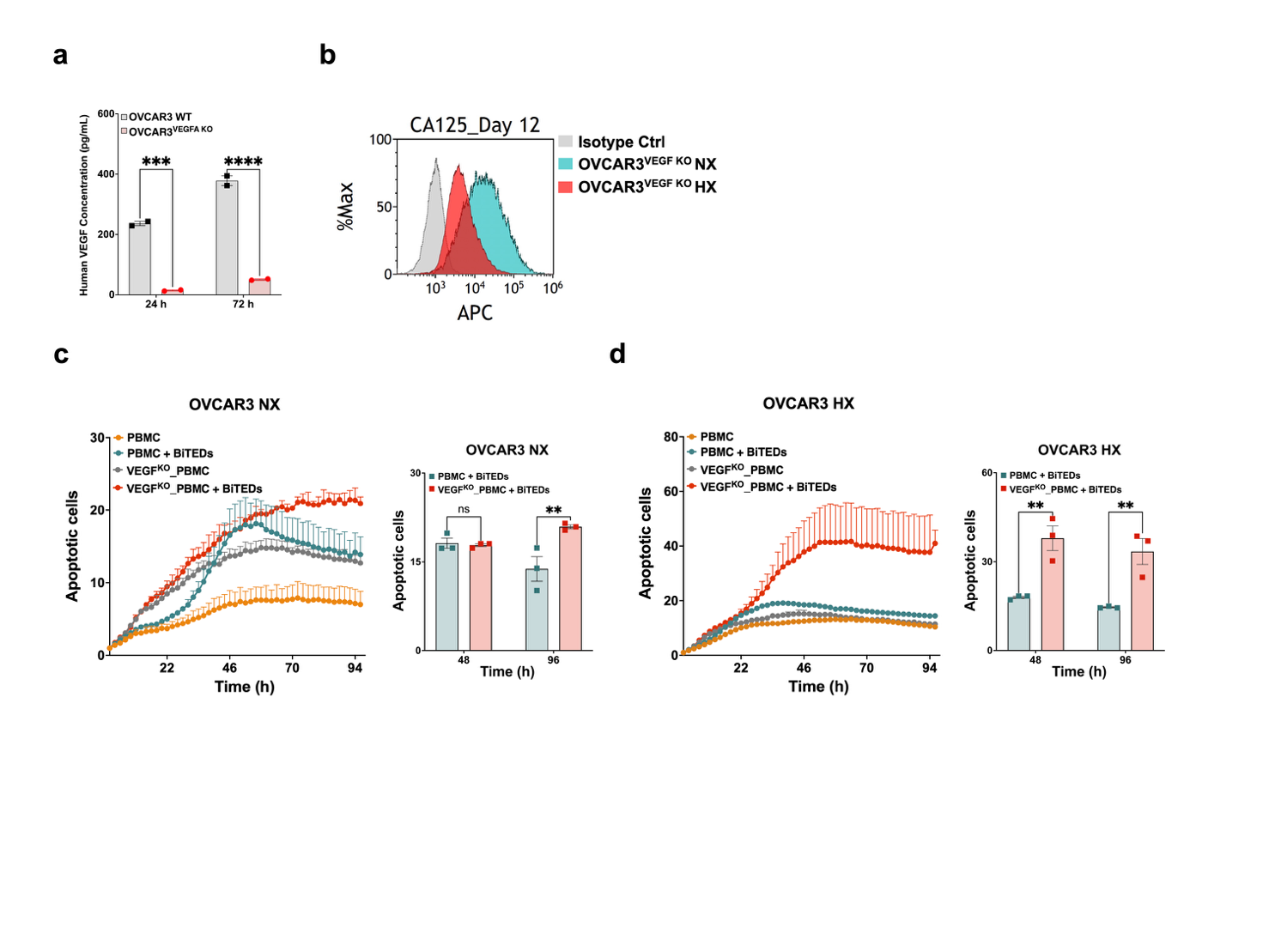


**Supplementary Figure 8: Evaluation of the effect of VEGF knockout on cytotoxicity**

(**a**). Evaluation of VEGF secretion in OVCAR3 cells with VEGF knocked out (OVCAR3^VEGF KO^) and control OVCAR3 cells at the indicated time points. (**b**). CA125 expression on OVCAR3^VEGF KO^ after 12 days under NX or HX conditions. MUC16-BITED cytotoxicity in OVCAR3^VEGF KO^ NX (**c**), and HX (**d**) conditions. All data are expressed as the means ± SEM from two independent measurements for panel a, and three independent measurements for panel c and d, and the differences between the groups were analyzed using the student’s t-test. ns, not significant; **p<0.01; ***p<0.001; ****p<0.0001.

Figure. S9.


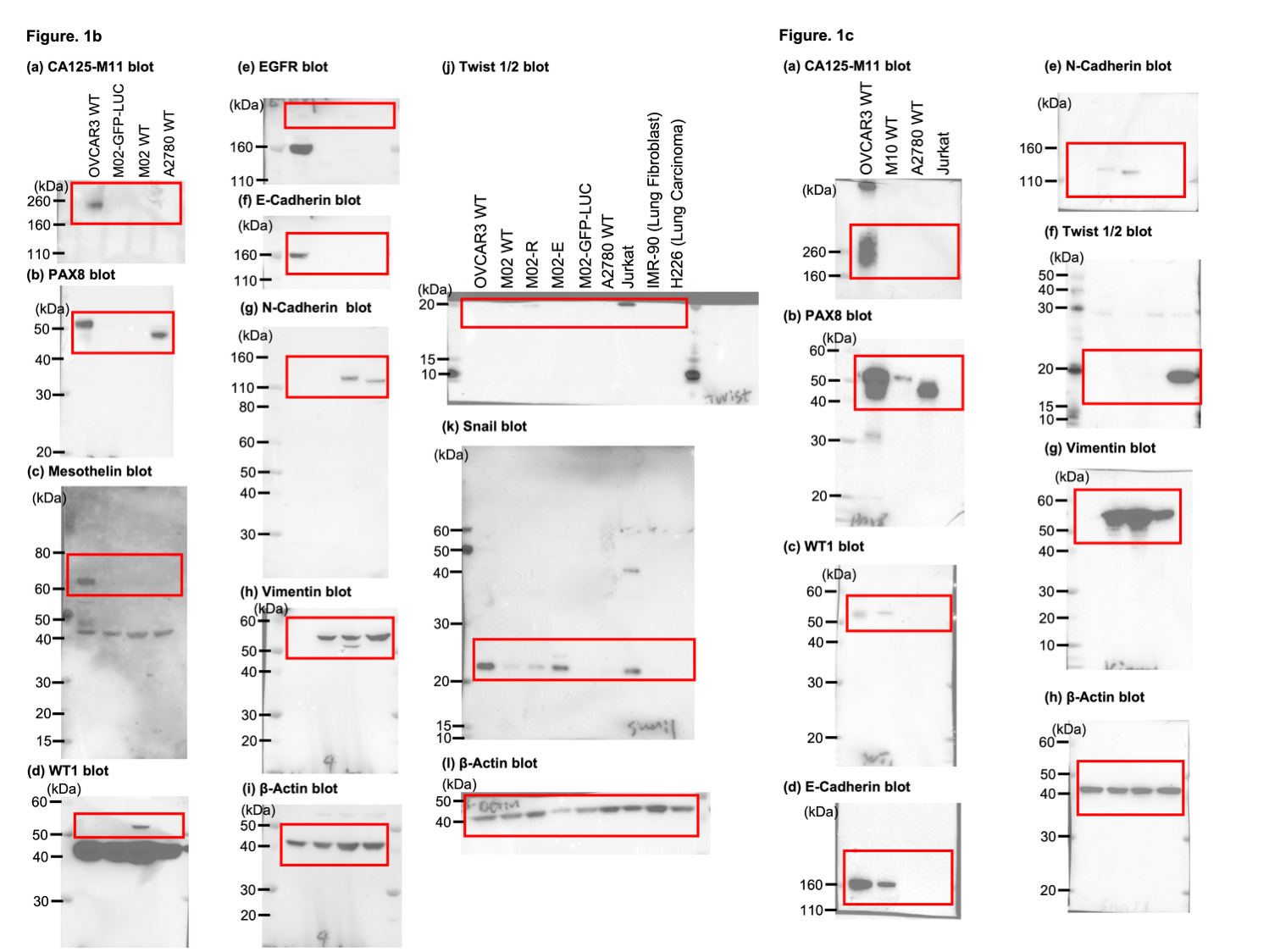


**Supplementary Figure 9: Original Western blot data corresponding to Figures 1b and 1c**

Red boxes indicate the cropped areas used in the main figures. Molecular weight markers (kDa) and sample labels are shown for reference.

Figure. S10.


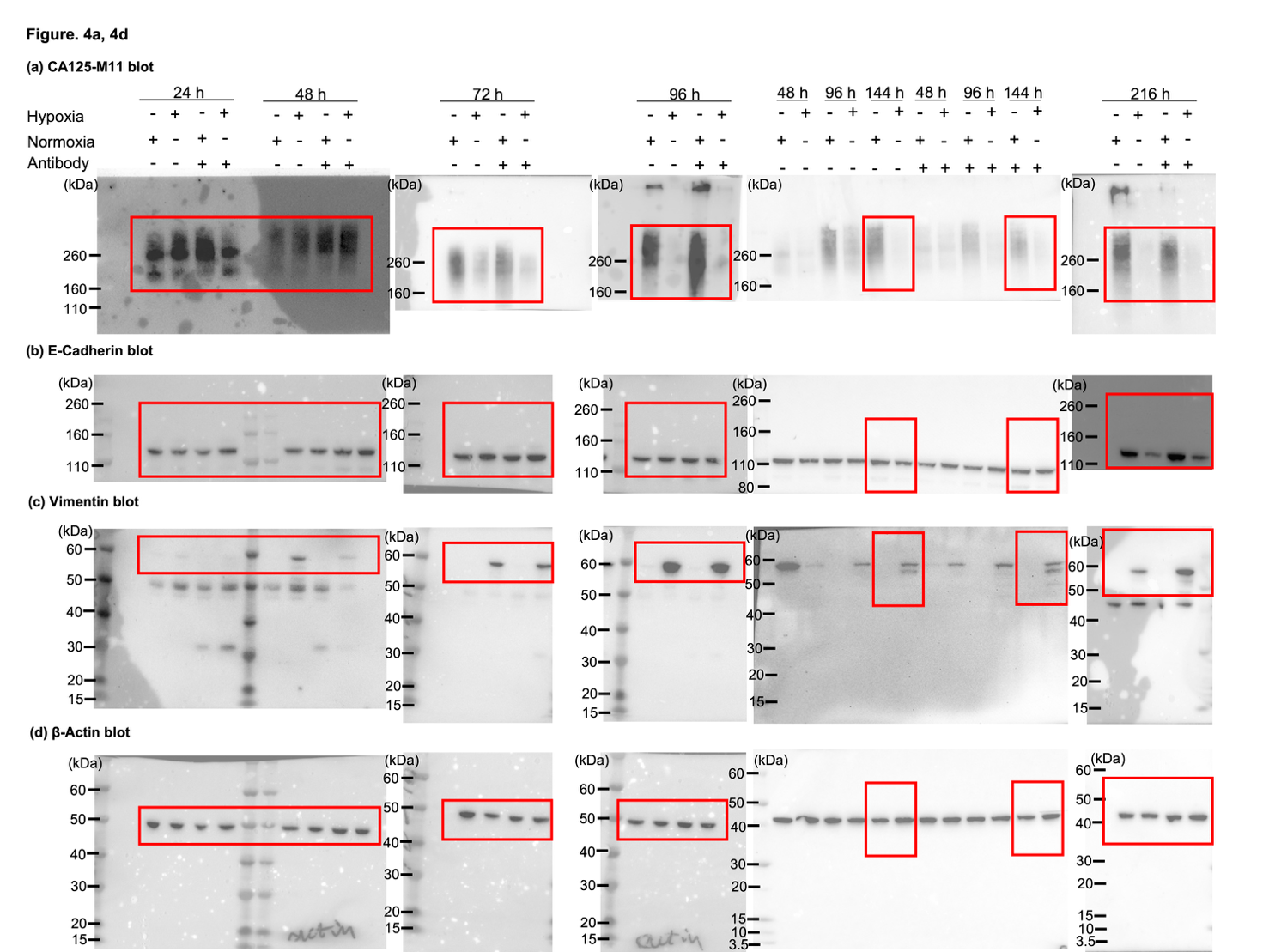


**Supplementary Figure 10: Original Western blot data corresponding to Figures 4a and 4d**

Red boxes indicate the cropped areas used in the main figures. Molecular weight markers (kDa) and sample labels are shown for reference.

Figure. S11.


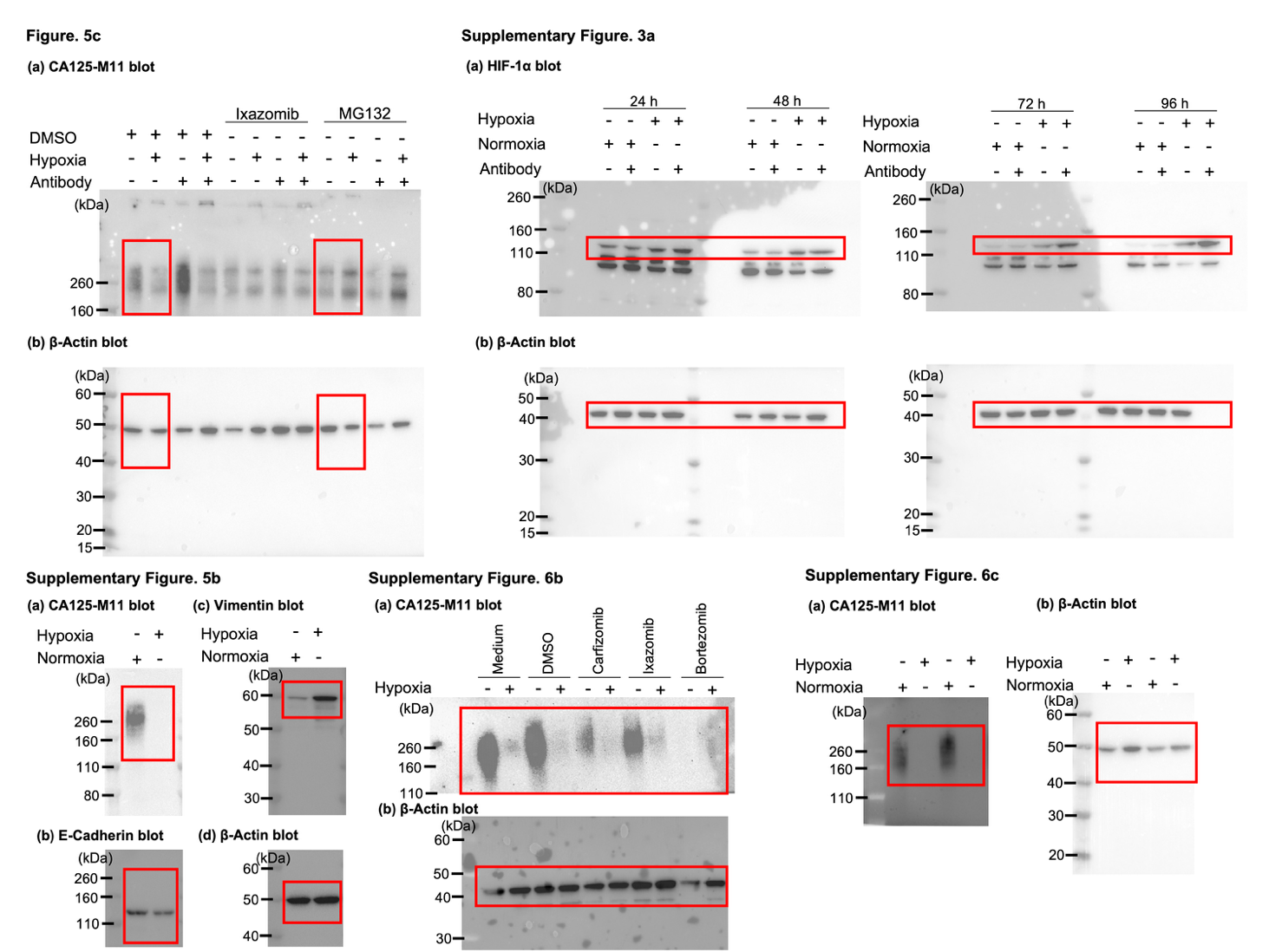


**Supplementary Figure 11: Original Western blot data corresponding to Figure 5c,**

**Supplementary Figures 3a, 5b, 6b and 6c**

Red boxes indicate the cropped areas used in the main figures. Molecular weight markers (kDa) and sample labels are shown for reference.

Table S1.

**Supplementary Table 1: MFI for Multiplex Cytokine and Chemokine analysis**

| Avg Net MFI |  |  |  |  |  |  |  |  |
| --- | --- | --- | --- | --- | --- | --- | --- | --- |
| Sample | SDF-1 alpha | IL-27 | IL-1 beta | IL-2 | IL-4 | IL-5 | IP-10(CXCL10) | MIP-1 alpha(CCL3) |
| Standard1 | 3134.75 | 16391.25 | 10173.75 | 4818.5 | 16756.5 | 4151 | 15597 | 14324.25 |
| Standard2 | 2809.75 | 7737 | 5510.5 | 3957 | 15624.75 | 4920 | 14192.75 | 13841.5 |
| Standard3 | 1640 | 2103.5 | 2066.75 | 2190.5 | 7800.75 | 2888.5 | 5331 | 13709.25 |
| Standard4 | 739.75 | 585.75 | 725.25 | 802 | 3070.75 | 907.25 | 1706 | 9359.5 |
| Standard5 | 180.25 | 122.25 | 184.5 | 125 | 522 | 159.25 | 280.5 | 2759.75 |
| Standard6 | 50 | 28.5 | 49.5 | 18 | 109 | 36 | 45.75 | 651.75 |
| Standard7 | 4.25 | 6 | 10.5 | 3 | 27 | 7 | 7.5 | 129.25 |
| P1 | 463.75 | -5 | -3.5 | -3 | -2.75 | 0 | 520.25 | 26.75 |
| P2 | 427 | -5.5 | -3 | -1.5 | 7.75 | 0 | 515.75 | 102.75 |
| P2P | 1238.25 | -5 | -2.5 | 1 | 81.75 | 8.25 | 4803.5 | 274.75 |
| P2T | 836.5 | -5 | -2 | 0 | 92.25 | 11.5 | 6498.5 | 155.5 |
| P4 Pre | 265 | -4 | -3.5 | 8 | -2.75 | 2.5 | 122 | 46.5 |
| P4 Post | 347.25 | -4.5 | -3 | -0.5 | 0.75 | 0 | 457.75 | 70.5 |
| P5 Pre | 493.25 | -5 | -2.5 | -2 | -1.25 | 0.25 | 282.5 | 296.5 |
| P5 Post | 435.75 | -4.5 | 6.5 | 0 | 7.75 | 1.5 | 737.25 | 864.75 |
| P6 | 414.25 | -3.5 | -2 | -1.5 | 0.25 | 1 | 813 | 6.25 |
| P7 Pre | 323.75 | -4.5 | -2.5 | -1 | -6 | 0 | 77.5 | -19.25 |
| P7 Post | 279 | -0.5 | 3.5 | 4.5 | 23.75 | 6 | 834.5 | -2.25 |
| P8 | 441.75 | -3.25 | -2 | -1.5 | -1.75 | 0.5 | 632 | 192.75 |
| P9 | 293.25 | -4 | -2.5 | -1.5 | -3 | 0.5 | 279.25 | 2.75 |
| P10 | 749.25 | -3.75 | -2 | 1 | -0.25 | 2.25 | 290.75 | 284.25 |
| P10 SN | 1188.75 | 5.5 | 5 | 7.25 | 159.25 | 8.75 | 12119 | 4054.25 |
| P12 | 549.75 | -4 | -2.5 | 167.25 | 4.5 | 27.5 | 282.75 | 159.25 |
| P13 | 405.25 | -4.5 | -2 | -2 | -4.25 | 0 | 178.25 | -3.25 |
| D1 | 399.75 | -3.5 | -2.5 | -1 | -3.5 | 1.5 | 93.5 | 25.25 |
| D2 | 500.25 | -4 | -0.5 | 0 | 2 | 2.5 | 144.25 | 30.25 |
| D3 | 509.25 | -4.25 | -3 | 0 | -4.75 | 1.5 | 129.25 | 30.25 |
| RPMI | -22 | -1.5 | -1.5 | 0.5 | -4.25 | 1.5 | -0.75 | -21.5 |
| P2P SN | 1066 | 2.5 | 5 | 129.25 | 93 | 39.25 | 3812 | 188.75 |
| P2T | 637.75 | -2 | 1.5 | 38.5 | 88 | 25.5 | 4037.75 | 65.75 |
| P10 SN | 964.5 | 49.75 | 56.75 | 600 | 257.75 | 254.75 | 7452.5 | 4316 |
| M1 | -6.25 | -0.75 | 0 | -0.25 | 2.5 | 0.75 | 832 | 567.5 |
| M2 | 47.75 | 14 | 165.75 | 57 | 121.75 | 24.5 | 15332.75 | 10994.5 |
| M3 | 172.75 | 6 | 168.5 | 29 | 157.25 | 29.25 | 13156 | 11016.25 |
| M4 | 19.5 | 0.5 | 2.5 | 2.25 | 13008.25 | 2 | 112.25 | 1287.75 |
| M5 | 35.25 | 0 | 0 | 1.25 | 4.75 | 2.5 | 539.5 | 868.5 |
| M6 | 168 | 9 | 153.25 | 37.75 | 156 | 32.25 | 14204 | 11002.75 |
| M7 | 154 | 7 | 216.5 | 19.5 | 141.25 | 26.25 | 12417.25 | 8483.75 |
| M8 | 5 | -0.5 | 3 | 2.75 | 12654.25 | 2 | 577.75 | 4940.75 |
| S1 | 2295.75 | 17681 | 10976.5 | 4197 | 14862.25 | 2729.5 | 13544.25 | 13236.75 |
| S2 | 1689 | 7648.25 | 4605.25 | 3605.5 | 10580.25 | 2753 | 14511.25 | 13021.25 |
| S3 | 1032 | 2399.75 | 1632.25 | 2110.25 | 6011 | 3276.5 | 6849 | 13304.5 |
| S4 | 392.75 | 575.5 | 503.25 | 666.5 | 1848.25 | 1274.25 | 1764.5 | 9731.25 |
| S5 | 89.5 | 110 | 148 | 129.75 | 321 | 269.5 | 381 | 3311.75 |
| S6 | 2.75 | 21.25 | 30 | 14.75 | 36.75 | 46.5 | 42 | 693.25 |
| S7 | -16.75 | 1.5 | 7 | 2 | 4.25 | 11.75 | 3 | 94.5 |
| S8 | -22.75 | -2 | -1 | 0.75 | -4.25 | 1 | -0.75 | -25.75 |

| Avg Net MFI |  |  |  |  |  |  |  |  |
| --- | --- | --- | --- | --- | --- | --- | --- | --- |
| Sample | IL-6 | IL-7 | IL-8(CXCL8) | IL-10 | Eotaxin(CCL11) | IL-12p70 | IL-13 | IL-17a (CTLA-8) |
| Standard1 | 8586.25 | 18562.25 | 18201.5 | 11409 | 975.5 | 23953.5 | 10246.25 | 5506 |
| Standard2 | 8092.5 | 9462.5 | 14947.5 | 6098.5 | 13671.25 | 21346 | 6389 | 3235.5 |
| Standard3 | 4292.25 | 1884 | 6753.5 | 1697.75 | 5797 | 10563 | 2135 | 626.25 |
| Standard4 | 1010.25 | 409 | 2556.25 | 526 | 1905.5 | 4743.5 | 452 | 144.75 |
| Standard5 | 92.5 | 71 | 553.75 | 127.75 | 255 | 1389 | 41 | 26.5 |
| Standard6 | 12.25 | 15.5 | 126.75 | 28.5 | 37.5 | 368.75 | 2.75 | 7 |
| Standard7 | 2.25 | 3 | 35.5 | 7.25 | 6.75 | 89 | 0 | 1.5 |
| P1 | -1.75 | 0.5 | -8 | -7.25 | 0 | 3.5 | -4 | -2 |
| P2 | 2.75 | 4.75 | 189.75 | 6.75 | 1410.75 | 0.75 | -3.5 | 0.75 |
| P2P | 3194 | 1.5 | 2952.5 | 117.75 | 1008.5 | 2 | -2.5 | 1.5 |
| P2T | 1680 | 0 | 521 | 36.75 | 431 | 1 | -2 | 1 |
| P4 Pre | 18.25 | 0.75 | -10 | -4.75 | 1228.25 | -1.5 | -3 | -2 |
| P4 Post | -0.25 | 1.5 | -2 | -3.75 | 1441.25 | -1 | -2.5 | -2.25 |
| P5 Pre | -0.75 | 1.75 | 84 | -7.25 | 1946 | -1 | -2 | -2.5 |
| P5 Post | 3570 | 8 | 641.25 | -4.75 | 3728 | -0.5 | -2 | -2 |
| P6 | 2.25 | -0.25 | -6 | -7.75 | 11.5 | 0 | -2.5 | -1.5 |
| P7 Pre | -1.25 | 5 | -15.5 | -5.75 | 1828.25 | -0.75 | -2.75 | -1 |
| P7 Post | 13.25 | 8 | 60.5 | 0.25 | 187 | 5.25 | 1.5 | 2.75 |
| P8 | -1.75 | 26.5 | 52.25 | -4.25 | 2116.75 | 0 | -2 | -0.75 |
| P9 | -0.75 | 8 | -10 | -6.25 | 740 | 1 | -2.5 | -1 |
| P10 | 6.75 | 8.75 | 74.5 | 2.75 | 1948 | 1 | -1.5 | 2 |
| P10 SN | 7575.25 | 23.5 | 11612.5 | 381.25 | 6040.25 | 11.75 | 0.5 | 14.5 |
| P12 | 149.25 | 30.5 | 17.75 | -4.5 | 2503 | -0.5 | -2.5 | 2 |
| P13 | -0.75 | 5.75 | -15.75 | -8.25 | 972.75 | -1 | -3 | -1.5 |
| D1 | -1.25 | 22.75 | -13 | -6.75 | 1396 | 0 | -2.25 | -1 |
| D2 | 4.75 | 26.5 | -7.75 | -5.75 | 2259.5 | 1 | -2.5 | 1.25 |
| D3 | 0.25 | 15.5 | -10.25 | -6.75 | 2539.25 | 2 | -2.5 | -1 |
| RPMI | -0.75 | 1.25 | -19 | -4.25 | -2.5 | 5 | -1.5 | -0.5 |
| P2P SN | 3987.75 | 11.25 | 1718 | 335 | 1043 | 10.5 | 1.25 | 12 |
| P2T | 2050.75 | 6.25 | 249.5 | 108.5 | 272.75 | 5.25 | 1 | 4 |
| P10 SN | 5218.75 | 199.75 | 9855.5 | 607 | 4490.5 | 62.5 | 54.75 | 91.25 |
| M1 | 3.25 | 2 | 9655 | -0.5 | 6.25 | 2 | -0.5 | 4.5 |
| M2 | 9190.5 | 13.5 | 13183.75 | 3496.25 | 28.25 | 1805 | 5 | 21.25 |
| M3 | 8846.5 | 15 | 12413.5 | 12950.25 | 86 | 38.25 | 1.5 | 15.75 |
| M4 | 0.75 | 5.75 | 7071 | 13.25 | 26 | 9.5 | 3 | 4.5 |
| M5 | 0.25 | 3.5 | 10075 | 6.25 | 45 | 3 | -0.5 | 4.5 |
| M6 | 9169 | 14.5 | 12867 | 3219 | 77 | 2351.5 | 5 | 19 |
| M7 | 8782.5 | 14.25 | 11668.25 | 11913.25 | 66.25 | 33.25 | 3 | 16.75 |
| M8 | 0.25 | 4.5 | 7380 | 11.25 | 28.25 | 10 | 2 | 5 |
| S1 | 7969 | 19508.5 | 15027.75 | 12654.75 | 643 | 23884.5 | 10526.25 | 4363.75 |
| S2 | 7977.75 | 11059 | 8621.5 | 8084 | 285.5 | 21970.75 | 5091.25 | 3240 |
| S3 | 5954.25 | 3411 | 3836 | 3216.5 | 7008.75 | 12733.25 | 1313 | 1172.5 |
| S4 | 1708.5 | 653.5 | 1008 | 890 | 2013.75 | 5336.5 | 169.5 | 217.75 |
| S5 | 353.75 | 121 | 164 | 233 | 366.5 | 1844.25 | 13 | 45 |
| S6 | 21.25 | 22.75 | 17.5 | 48.25 | 31.5 | 451.25 | 1 | 7 |
| S7 | 3.25 | 5 | -12 | 7.25 | 1.25 | 120.5 | -0.5 | 1 |
| S8 | -1.25 | 1 | -20.75 | -4.25 | -2 | 4.5 | -1 | -1.5 |

| Avg Net MFI |  |  |  |  |  |  |  |  |
| --- | --- | --- | --- | --- | --- | --- | --- | --- |
| Sample | IL-31 | IL-1RA | RANTES(CCL5) | IFN gama | GM-CSF | TNF alpha | MIP-1 beat(CCL4) | IFN alpha |
| Standard1 | 17089.25 | 5201.5 | 13221.5 | 16243 | 15696.75 | 9490.5 | 12086.5 | 22531.5 |
| Standard2 | 12935.25 | 2881.25 | 15405.75 | 16448.75 | 12212 | 5556.75 | 9079 | 16139.75 |
| Standard3 | 6959.25 | 790.25 | 13656.75 | 9036.25 | 4611.75 | 2009.5 | 5758.5 | 6670.25 |
| Standard4 | 2413.5 | 210.25 | 6167.5 | 3684.75 | 728.5 | 669.5 | 3419.5 | 2305.5 |
| Standard5 | 494 | 47 | 969 | 866.25 | 53.25 | 157.5 | 985.5 | 503.5 |
| Standard6 | 115 | 8.25 | 168.5 | 230 | 7.75 | 35.75 | 228.75 | 111.75 |
| Standard7 | 23 | 17.25 | 31.75 | 62 | 1 | 8.5 | 42 | 26.5 |
| P1 | -33.5 | -59 | 1311 | -5 | -2.5 | 0.5 | 330.75 | 22.25 |
| P2 | -32.5 | -75.25 | 1568.75 | -5 | -1.5 | 2.5 | 775 | 16.5 |
| P2P | -29 | -38.75 | 380.75 | 4 | 1 | 8 | 960.25 | -0.5 |
| P2T | -30.5 | -40 | 415.5 | 3.5 | 0 | 5.5 | 749.5 | -4 |
| P4 Pre | -30 | -89.75 | 725.5 | -6.5 | 1 | 1.5 | 474.5 | 28 |
| P4 Post | -32 | -88.25 | 825.25 | -5.75 | -2 | 2 | 486 | 26.5 |
| P5 Pre | -31.5 | 25.5 | 759.5 | -3.25 | -3 | 1.5 | 763.5 | 50.75 |
| P5 Post | -31.5 | 321.25 | 1094.75 | 0.75 | -0.5 | 10.5 | 965 | 83 |
| P6 | -33 | -70.5 | 1969.25 | -1.5 | -1.5 | 4.5 | 166.5 | 18 |
| P7 Pre | -32 | -90.25 | 496 | -7.75 | -2 | 2.75 | 504.5 | 36.75 |
| P7 Post | -25.5 | -76.75 | 5630.25 | 3.5 | 1.5 | 3 | 431.5 | 40.75 |
| P8 | -30.75 | -89.25 | 1805.5 | -7.5 | -1.5 | 1.5 | 935.75 | 41.5 |
| P9 | -31.75 | -84.25 | 850.25 | -5 | -2 | 1.75 | 539.5 | 70.75 |
| P10 | -31.25 | -67.5 | 1023.25 | -4.5 | -0.5 | 7.5 | 895.5 | 27.75 |
| P10 SN | -11.75 | 56.75 | 1363 | 107.5 | 5 | 29.75 | 2728.25 | 21.75 |
| P12 | -18 | -75.75 | 704 | -6 | 37 | 6.5 | 760.25 | 49 |
| P13 | -32.5 | -83.25 | 684 | -8.5 | -1.5 | 1 | 148.5 | 3 |
| D1 | -31 | -66.75 | 923.5 | -6.5 | -1 | 3.75 | 650.25 | 41 |
| D2 | -30 | -72.75 | 1373 | 3 | 3 | 9.25 | 624.5 | 48 |
| D3 | -30 | -79.5 | 1181.5 | -6 | -0.75 | 3 | 686.25 | 47 |
| RPMI | -26 | -61 | -25 | -7.5 | 0 | 1.5 | -19.25 | -6.5 |
| P2P SN | 9 | 104 | 326 | 57 | 36 | 22 | 799.75 | 63.5 |
| P2T | -23.5 | 42.5 | 329 | 38 | 14.5 | 13.5 | 574.5 | 15.5 |
| P10 SN | 468.75 | 207 | 1126.75 | 209 | 283 | 85.75 | 2530 | 245.5 |
| M1 | -28 | 643.5 | 3.25 | -3.5 | 0.5 | 11.75 | 300.75 | -4 |
| M2 | -23 | 3905 | 6804.75 | 10266.5 | 54.75 | 4712.25 | 9779.5 | 103 |
| M3 | -18.25 | 5697.25 | 7411 | 3 | 29.75 | 3032.25 | 10775 | 146.5 |
| M4 | -26.25 | 3465 | 22 | -0.5 | 392.5 | 56.25 | 771.5 | -2.5 |
| M5 | -24.75 | 2565.25 | 35 | -4 | 2.5 | 7 | 558.75 | -0.5 |
| M6 | -20 | 4835.25 | 7179 | 10323.25 | 53.5 | 5287.5 | 10076 | 101.75 |
| M7 | -21.25 | 5530.25 | 7188.5 | 3 | 22 | 2558 | 9745.5 | 144.5 |
| M8 | -26 | 3609.25 | 46.75 | 0.5 | 356.5 | 56.75 | 1674 | -1.75 |
| S1 | 17710.75 | 4367.75 | 10902.75 | 14018.75 | 15136.5 | 9223.75 | 10930.25 | 18954.25 |
| S2 | 7091 | 2215.5 | 8030.75 | 10918 | 13324.25 | 7328 | 7630.5 | 10613.5 |
| S3 | 2941.75 | 637.75 | 7913.5 | 5746.25 | 7731.5 | 2600.25 | 4613 | 4170.25 |
| S4 | 702 | 64.75 | 3553.5 | 1637 | 1646.25 | 891 | 2456 | 814 |
| S5 | 141.25 | -30.5 | 555.5 | 447.5 | 127.75 | 240 | 565 | 104.25 |
| S6 | 4.25 | -53.5 | 38.5 | 85 | 12 | 57 | 56.25 | 7.25 |
| S7 | -17.5 | -59.25 | -18 | 14 | 2.5 | 14 | -2.75 | -3.75 |
| S8 | -26.5 | -60.75 | -24.5 | -8 | 0.5 | 2 | -19.5 | -6 |

| Avg Net MFI |  |  |  |  |  |  |  |  |
| --- | --- | --- | --- | --- | --- | --- | --- | --- |
| Sample | MCP-1(CCL2) | IL-9 | TNF beta | GRO alpha(CXCL1) | IL-1 alpha | IL-23 | IL-15 | IL-18 |
| Standard1 | 24763 | 9728.25 | 11828 | 2164.25 | 20729 | 16410 | 22152.75 | 2209.5 |
| Standard2 | 23935 | 6405.75 | 10324 | 23795.5 | 11449.25 | 9354 | 17231.25 | 6210.5 |
| Standard3 | 10972.25 | 2499 | 6092.75 | 15622 | 8586.25 | 3667 | 6877.25 | 2864.75 |
| Standard4 | 3482.5 | 783 | 2797.5 | 6115 | 3380.5 | 1322.75 | 1090.5 | 983.5 |
| Standard5 | 379.25 | 151.75 | 820.5 | 1136.5 | 598 | 318.75 | 62.5 | 178.5 |
| Standard6 | 41 | 30.75 | 192.5 | 185.75 | 130 | 74.25 | 8 | 37 |
| Standard7 | 6.5 | 10.75 | 59 | 24.25 | 29.5 | 13 | 0.5 | 11 |
| P1 | 61.25 | -25.75 | -175 | 13.5 | 9.25 | -48 | -2.5 | 0 |
| P2 | 44 | -13.75 | -172.5 | 680.5 | 8.75 | -46 | -1 | 17 |
| P2P | 181 | 17.75 | -160.5 | 4846.5 | 16.5 | -44 | 2.5 | 22.25 |
| P2T | 117.25 | -14.75 | -155.75 | 926.75 | 2.5 | -35.5 | 1 | 21 |
| P4 Pre | 53.25 | -14 | -173 | 21.5 | 0.5 | -45.5 | 31 | 4 |
| P4 Post | 66.25 | -22.25 | -173.5 | 34.5 | 0.5 | -48 | 2 | 11 |
| P5 Pre | 178.25 | -21.25 | -173 | 24.5 | -1 | -46.75 | 0.5 | 1.5 |
| P5 Post | 2541 | -4.5 | -172.25 | 212.5 | 0 | -46.5 | -0.5 | 12 |
| P6 | 236 | -23 | -171.5 | 23.75 | 13.5 | -46.5 | 0 | 10.25 |
| P7 Pre | 56.75 | -23 | -174 | 18.5 | 2.5 | -47.5 | -1 | 1.5 |
| P7 Post | 137 | -12.75 | -157.75 | 22 | 6.75 | -30.25 | 7 | 42 |
| P8 | 116.5 | -21.75 | -169 | 170.5 | 0.5 | -46.5 | 0 | 2.25 |
| P9 | 239.25 | -24.25 | -172 | 62 | 1.5 | -47.5 | 1 | 11.5 |
| P10 | 213.5 | -20.5 | -170.5 | 53.75 | 18.5 | -45.5 | 1 | 6.25 |
| P10 SN | 20462.75 | 207.25 | -90.25 | 6593.25 | 12 | 16.5 | 11.25 | 64 |
| P12 | 107.75 | 19.75 | -168.5 | 61.5 | 81.25 | -41.5 | 169 | 2 |
| P13 | 22.75 | -25.25 | -174 | 12.5 | 10.75 | -47 | -1 | -1.5 |
| D1 | 178.25 | -23.75 | -172 | 82 | 2.5 | -46 | 0.5 | 3.5 |
| D2 | 270 | -18.75 | -170.5 | 95.5 | 28 | -45.75 | 3.5 | 7.5 |
| D3 | 156.25 | -21.75 | -171.5 | 104.75 | 2.5 | -45.5 | 1.5 | 6.25 |
| RPMI | 1 | -18.25 | -157 | 1.25 | 0 | -37.25 | 1 | -1 |
| P2P SN | 417 | 278.25 | -147 | 7028.5 | 17.25 | -31.25 | 315 | 34 |
| P2T | 145 | 41.25 | -148.75 | 1015.75 | 3.5 | -41 | 81.75 | 23.5 |
| P10 SN | 16686.75 | 767.25 | 45 | 4653.25 | 27.5 | 249.5 | 645 | 109.25 |
| M1 | 19943.25 | 181.25 | -159.75 | 173.25 | 23.5 | -38.5 | 0.75 | 4 |
| M2 | 21613.25 | 374 | -63.5 | 16105.75 | 150 | -9 | 9.75 | 151 |
| M3 | 21812.75 | 387.75 | -112 | 23074.5 | 34 | -11 | 7 | 46.75 |
| M4 | 20067.25 | 92.75 | -146.25 | 2565.75 | 2.5 | 84.5 | 4 | 47.25 |
| M5 | 21378.25 | 181.25 | -144.25 | 6454.25 | 2.5 | -35.25 | 3.5 | 13.5 |
| M6 | 21830.75 | 350.25 | -60.25 | 20248.25 | 57.5 | -10.25 | 11.5 | 398.5 |
| M7 | 17638.75 | 440 | -119.5 | 22519.5 | 37 | -11 | 8.5 | 50 |
| M8 | 21408.25 | 110 | -139 | 2931.5 | 1.75 | 72.25 | 4.5 | 42.25 |
| S1 | 23964.5 | 9453.5 | 11133.5 | 1449 | 18153 | 11469.25 | 22452 | 2120.75 |
| S2 | 22896 | 6605.25 | 9680.75 | 11073.75 | 11420.5 | 5408.25 | 17380.5 | 1867 |
| S3 | 11071.25 | 2985.75 | 6460.75 | 19086.25 | 7630.5 | 2195.75 | 8255.25 | 2541 |
| S4 | 3265.25 | 788.25 | 2276.5 | 6688.75 | 3424.75 | 636 | 1340.5 | 815 |
| S5 | 424.25 | 155.75 | 448 | 1554.75 | 775.75 | 127.25 | 69 | 165.5 |
| S6 | 33 | 16.75 | -15.75 | 229 | 161.5 | -7 | 10.5 | 32.5 |
| S7 | 4 | -11.25 | -125.75 | 24.25 | 34 | -27.5 | 2.75 | 7 |
| S8 | 1 | -17.5 | -157 | 1.5 | 0 | -36 | 1.5 | -1.5 |

| Avg Net MFI |  |  |
| --- | --- | --- |
| Sample | IL-21 | IL-22 |
| Standard1 | 8890 | 6798.25 |
| Standard2 | 6634.75 | 5830.75 |
| Standard3 | 4015.75 | 4188.75 |
| Standard4 | 741 | 2102.75 |
| Standard5 | 129.25 | 518.25 |
| Standard6 | 40 | 127 |
| Standard7 | 10.75 | 38.75 |
| P1 | -10 | -29.75 |
| P2 | 3.5 | -7.75 |
| P2P | 6.75 | 9.75 |
| P2T | -2.5 | -8.75 |
| P4 Pre | -8.25 | -30.75 |
| P4 Post | -6.5 | -31.25 |
| P5 Pre | -5 | -22.25 |
| P5 Post | -2 | -18.25 |
| P6 | -8 | -22.5 |
| P7 Pre | -9 | -30.75 |
| P7 Post | 2 | -13.5 |
| P8 | 4.25 | -3.5 |
| P9 | -10.5 | -34.25 |
| P10 | 28.5 | 65.25 |
| P10 SN | 24 | 31.75 |
| P12 | -0.5 | -23.75 |
| P13 | -10.5 | -29.75 |
| D1 | -6.5 | -27.75 |
| D2 | -5.5 | -21.25 |
| D3 | -4.5 | -21.75 |
| RPMI | -6.5 | -22.5 |
| P2P SN | 35.75 | 38.5 |
| P2T | 5 | 3.75 |
| P10 SN | 95.75 | 122.75 |
| M1 | 4 | -13.75 |
| M2 | 45.25 | 50.25 |
| M3 | 49 | 128.25 |
| M4 | 7 | -13.5 |
| M5 | 5 | -13.25 |
| M6 | 41 | 49.25 |
| M7 | 47.75 | 122.75 |
| M8 | 9.5 | -8.25 |
| S1 | 7865 | 5773 |
| S2 | 5010.75 | 4129.75 |
| S3 | 2862 | 3039.75 |
| S4 | 769.5 | 1643.25 |
| S5 | 153.5 | 487.25 |
| S6 | 32 | 86.5 |
| S7 | 1.25 | 3.5 |
| S8 | -7 | -21.75 |

Table S2.

**Supplementary Table 2: Sequences of the primers used for qPCR assay**

| **Gene** |  | **Sequence (5’ to 3’)** | **Application** |
| --- | --- | --- | --- |
| MUC1    MUC3A    MUC4    MUC15    MUC16    GAPDH | F  R  F  R  F  R  F  R  F  R  F  R | TGCCGCCGAAAGAACTACG  TGGGGTACTCGCTCATAGGAT  TGACTCCCACACCTGTAACC  GGGGTGAGAGTTCGTAGGCT  CGTTCTGGGACGATGCTGAC  GATGGCTTGGTAGGTGTTGCT  TATTCACTTCTATCGGGGAGCC  GGGAATGACTCGCCTTGAGAT  CCAGTCCTACATCTTCGGTTGT  AGGGTAGTTCCTAGAGGGAGTT  GGAGCGAGATCCCTCCAAAAT GGCTGTTGTCATACTTCTCATGG | qPCR    qPCR    qPCR    qPCR    qPCR    qPCR |

F, Forward primer; R, Reverse primer.
